# Network dynamics of human face perception

**DOI:** 10.1371/journal.pone.0188834

**Published:** 2017-11-30

**Authors:** Cihan Mehmet Kadipasaoglu, Christopher Richard Conner, Vatche George Baboyan, Matthew Rollo, Thomas Allyn Pieters, Nitin Tandon

**Affiliations:** 1 Vivian Smith Department of Neurosurgery, Univ. of Texas Medical School at Houston, Houston, Texas, United States of America; 2 Memorial Hermann Hospital, Texas Medical Center, Houston, Texas, United States of America; Centre de neuroscience cognitive, FRANCE

## Abstract

Prevailing theories suggests that cortical regions responsible for face perception operate in a serial, feed-forward fashion. Here, we utilize invasive human electrophysiology to evaluate serial models of face-processing via measurements of cortical activation, functional connectivity, and cortico-cortical evoked potentials. We find that task-dependent changes in functional connectivity between face-selective regions in the inferior occipital (f-IOG) and fusiform gyrus (f-FG) are bidirectional, not feed-forward, and emerge following feed-forward input from early visual cortex (EVC) to both of these regions. Cortico-cortical evoked potentials similarly reveal independent signal propagations between EVC and both f-IOG and f-FG. These findings are incompatible with serial models, and support a parallel, distributed network underpinning face perception in humans.

## Introduction

Converging evidence from behavioral, functional, and lesional data has identified a subset of cortical regions, biased towards the right hemisphere, that are believed to form a distributed network for face perception[1–4]. Current insights into the neural basis of face perception are derived from studies of brain-lesions[5–12] and single neuron recordings [13–15] in the monkey inferotemporal cortex (IT) (for review see [16, 17]). More recently, electrophysiological studies in non-human primates have identified as many as six, widely distributed face-selective neuronal clusters[18]–from the superior temporal sulcus (STS; upper and lower) to anterior IT[19–23].

In humans, a distributed representation for face processing was first suggested by lesional studies of patients with the inability to perceive and/or recognize faces (i.e. prosopagnosia) [4, 24–26]. Post-mortem studies of these individuals revealed that their lesions varied in location, from the occipital to the temporal pole, but were also generally biased towards the right hemisphere[2, 3, 26]. Evidence for the distributed and asymmetric cortical representation of face processing was supported in healthy human subjects following the advent of non-invasive neuroimaging technology in the 1990s –positron emission tomography and functional magnetic resonance imaging (fMRI)[27, 28]. However, as fMRI became the dominant human neuroimaging technique, studies began to report almost exclusively on three regions presenting with the largest face-selective activity: the STS (f-STS), inferior occipital and fusiform gyri (f-IOG and f-FG, respectively) [23, 26, 29–33].

In 2000, a highly influential model of face processing was proposed by Haxby and colleagues[1, 34], which codified these three regions into a core system for the visual processing of faces [1, 3, 30]. Concurrently, focus began to broaden from which brain regions were activated, towards how these distributed brain regions might interact during face perception. Building on prior feed-forward, hierarchical visual [35, 36] and cognitive[37] models, it was argued that face processing should follow similar principles–i.e. occur in stages of increasing complexity along a postero-anterior axis in the ventral visual cortex. Thus, feature detection (e.g. eyes, mouth, nose) was expected to precede facial representation (a complete face), which in turn should precede facial recognition (identity)[1, 38]. However, while studies had thus far identified potentially distinct functions for the f-FG (invariant aspects of face perception, identity) and the f-STS (changeable aspects of face perception, gaze and expression), the f-IOG’s function at that time was still largely unknown[1]. Haxby and colleagues proposed that the relatively posterior anatomical location of the f-IOG made it a likely candidate for early feature detection, as it was positioned to provide input to both the downstream f-FG and f-STS[1].

Since its proposal 16 years ago, Haxby’s hierarchical model has dominated the field of human face perception, and many studies have interpreted their findings in its context [3, 30, 34, 36, 39]. Recently, however, new evidence has emerged that questions the validity of such serial, hierarchical accounts [3, 4]. Prosopagnosic patients with uni- or bilateral f-IOG lesions demonstrate normal f-FG activation and behavioral performances matching healthy individuals during basic-level categorization tasks (for both real and ambiguous stimuli) [40]. If the hierarchical model were correct, however, the loss of f-IOG input should have precluded normal f-FG function [41, 42]. Instead, these individuals suffer from an inability to *differentiate* faces (i.e. identity discrimination) [40]. These findings led to the proposal of an alternative model of face processing that relies on parallel, distributed interactions between early visual cortex (EVC) and the f-IOG and f-FG [4]. According to the parallel model, f-FG detects faces independently of the f-IOG via direct EVC inputs that provide a coarse level of detail. Following detection, reentrant interactions between f-FG and f-IOG progressively refine facial representations to facilitate recognition. This parallel model drew inspiration from prior anatomical studies of monkey visual cortex[43], which had demonstrated that monkey visual regions were densely interconnected by a complex network of parallel, feedback, and re-entrant pathways[43–45].

To date, evidence from humans for either network model has come almost exclusively from non-invasive behavioral, functional, and stimulation studies. However, these approaches suffer from limited spatio-temporal resolution, and are ill-equipped to evaluate transient interactions between disseminated cortical regions [41, 46–48]. As such, the dynamics of information flow within the core face processing system remains a subject of continued debate[3, 4, 39]. And while human intracranial EEG (icEEG) recordings improve upon these limitations, they have focused principally on the functional response properties (timing/distribution/selectivity) of the core face network [49–56]. Thus far, a conclusive icEEG evaluation on the broader dynamics of the core face network has not been performed. Specifically, a primary tenet–that the f-IOG relays EVC input to the f-FG for the visual processing of invariant (i.e. static) face features–has not been validated using electrophysiological recordings [3, 4, 39].

Here, we investigate whether face perception invokes serial or parallel interactions between EVC and the f-IOG and f-FG. We note that the term “serial” is used as a natural contrast for the term “parallel”. It is not meant to imply a simplistic cortico-cortical pathway that serially and/or directly connects EVC to f-IOG to f-FG. Instead, “serial” is meant to represent the assumption that f-FG face processing is preceded by, and strictly depends upon, the f-IOG [3, 42, 57]. To accomplish these goals, we collected functional MRI (fMRI), icEEG, and cortico-cortical evoked potentials (CCEPs) data from 9 patients scheduled for subdural electrode implantation (RH *n =* 4; LH *n* = 5). Using the millisecond resolution of icEEG recordings, we evaluated task-dependent changes in high-frequency broadband gamma activity (BGA, 60–120 Hz) and compared the onsets of f-IOG and f-FG face-selectivity relative to each other during a visual face-naming task [47]. We then computed time-lagged measures of functional connectivity to estimate directed information flow between the EVC, f-IOG and f-FG [58, 59]. Lastly, we utilized CCEPs as a task-independent measure of electrophysiological connectivity [60] to evaluate neural signal propagation in cortico-cortical pathways between these three regions.

We hypothesize that if serial accounts (i.e. Haxby and colleagues[1]) are correct, functional connectivity should emerge first between EVC and f-IOG, and then between f-IOG and f-FG. Similarly, face-selectivity in the f-IOG should emerge prior to the f-FG, and electrical stimulation of EVC should evoke CCEP responses in f-IOG prior to f-FG. If instead parallel accounts of face-perception are correct, functional connectivity between EVC and f-FG should emerge prior to between f-IOG and f-FG connectivity; the f-FG should demonstrate face-selectivity no later than the f-IOG; and CCEP signal propagation latencies from EVC to both f-IOG and f-FG should not be significantly different.

## Materials and methods

fMRI, icEEG, and CCEP data were collected from 9 subjects (5 female, mean age 28 ± 8 years, mean IQ 96 ± 12) scheduled for right or left hemispheric sub-dural electrode implantation (RH *n* = 4; LH *n* = 5) to localize seizure onset sites. fMRI data were also collected from an independent group of 18 healthy volunteers (6 female, mean age 24 ± 4). All experimental procedures were reviewed and approved by the Committee for the Protection of Human Subjects (CPHS) of the University of Texas Health Science Center at Houston as Protocol Number: (HSC-MS-06-0385), and written informed consent was obtained from all subjects. All experiments were performed in accordance with relevant guidelines and regulations. Participant recruitment, both patient and healthy subject, took place over a time period from 2010 through 2016. From the 26 total participants included (9 patient and 18 healthy subject), all completed relevant study requirements and none dropped out.

### Experimental design

Subjects and healthy volunteers participated in a visual confrontation-naming task wherein images of famous faces were presented for the experimental condition and scrambled versions of the same face stimuli were presented as a low-level visual control. Face stimuli consisted of gray-scale, real images of famous individuals shown in frontal view with a grid overlay (celebrities, politicians, and historical figures taken from free online sources). Scrambled face control stimuli (referred to hereafter as “scramble”) were generated by rearranging the grid overlay so that low-level properties of the original face were preserved, while completely degrading any face-related information. High-level visual control stimuli were presented during a subsequent visual naming task using inanimate (tools and non-tool objects) and animate, non-face stimuli (animals and body-parts, hereafter referred to as “animate”). Animate and inanimate stimuli were taken from the standardized Snodgrass and Vanderwart’s object pictorial set [61].

fMRI and icEEG data were collected (separately) for both visual naming tasks. The data for non-face stimuli were used, in part, to identify electrodes localized over anatomically defined EVC, f-IOG, and f-FG regions, as part of a rigorous, multi-tiered inclusion criteria for this analysis (described below)[62].

### MRI

#### Cortical surface models

Pre-implantation anatomical MRI scans were collected using a 3T whole-body MR scanner (Philips Medical Systems, Bothell WA) equipped with a 16-channel SENSE head coil. Anatomical images were collected using magnetization-prepared 180 degree radio-frequency pulses and rapid gradient-echo (MP-RAGE) sequence, optimized for gray-white matter contrast, with 1 mm thick sagittal slices and an in-plane resolution of 0.938 x 0.938 mm [63]. Cortical surface models were reconstructed using FreeSurfer software (v5.1) [64], and imported to SUMA[65].

#### Functional MRI (fMRI) acquisition

Functional images were obtained with a gradient-recalled echo-planar imaging sequence (33 axial slices, 3 mm slice thickness, 2.75 in-plane resolution, 30 ms time echo, 2015 ms time repetition, flip angle 90°) [63]. Structural image processing, spatial transformations, functional image realignment, and statistical analyses were performed with AFNI [66]. Each fMRI volume was aligned to the skull-stripped high-resolution anatomical MRI using a registration algorithm with a mutual information cost function and bicubic resampling. The magnitude of each patient’s translational and angular head movements was inspected by examining the output realignment parameters to exclude data corrupted by gross motion artifact [63]. Gray matter volumes from each individual’s anatomical reconstruction were used to further constrain the fMRI data. No spatial smoothing was applied in further fMRI data analyses, and all visualizations were performed on the cortical surface to minimize topological inaccuracies [23].

#### fMRI localizer experiment

An fMRI face-localizer task was used to provide one (of three) independent criteria to identify face-selective electrodes for inclusion in this analysis (see below). fMRI experiments were performed by 4 of the 9 subjects schedule for electrode implantation (RH subjects 1–3 and LH subject 5), and 18 healthy volunteers. Due to clinical constraints (prior implantation of a vagus nerve stimulator to control epilepsy–which is not approved for echo-planar imaging), 5 subjects scheduled for electrode implantation were unable to participate in fMRI recordings (RH subject 4 and LH subjects 6–9), and were evaluated using grouped data from the healthy volunteer cohort.

Stimuli were presented in a block design. For each condition, 2 runs of fMRI data were collected that comprised 8 blocks (136 volumes per run), with each block comprising 10 task stimuli and 7 scrambled stimuli (20 s of task, and 14 s of scrambled control images). Data were collected for 160 individual stimuli each for category condition (face, and animate/inanimate) and for 224 stimuli of scrambled images (112 during each naming task) [63]. For the fMRI tasks, scrambled versions of animate/inanimate stimuli used were generated in the same fashion as for the scrambled face stimuli. Subjects and healthy volunteers were asked to internally (covertly) vocalize and respond with a button press for faces and animate/inanimate stimuli. For scrambled versions of the visual stimuli, subjects were instructed to press an adjacent button on the same controller using the thumb of the same hand.

Visual stimuli were presented at the onset of each functional image volume with Presentation software (version 11, Neurobehavioral Systems) using a screen positioned above the eyes (IFIS, Invivo; 1500 ms on-screen, 515 ms inter-stimulus interval, subtending a visual angle of ~10° x ~10°). Patient responses were monitored in real time using a fiber optic response pad connected to an interface unit (Current Designs), and by video monitoring of the patients face using a closed circuit television [63].

To identify higher responses to faces vs. other stimuli, BOLD response amplitudes and their corresponding *t*-values at each voxel time series were estimated using standard general linear model analyses (3dDeconvolve program in AFNI). Face-selective voxels were identified as faces > non-face stimuli (animate, inanimate, and scramble; t>3, p<0.01, voxel level) [23, 32, 67, 68].

For the 5 subjects unable to participate in the fMRI recordings (RH subject 4 and LH subjects 6–9), face-localizer data were derived from fMRI data recorded in the 18 healthy volunteers, using a surface-based, mixed-effects multilevel grouped analysis[69, 70]. Face-selective clusters from the grouped fMRI data were projected onto each subject’s native cortical surface separately to minimize anatomical shift errors. Face-selective voxels were identified as significant faces > non-face stimuli (animate, inanimate, and scramble; *q* < 0.05, corrected using family-wise error rate correction for multiple comparisons).

Although the use of normal subject data, transformed to individual patient brains, could be construed as problematic for use in electrode selection, this concern is ameliorated through two key methodological considerations: 1) the use of a surface-based co-registration approach that provides the precise alignment of inter-subject functional topology [65, 69, 71] [70] and 2) the fact that fMRI data provide only one (of three) distinct metrics used for electrode selection (see below).

### Intracranial EEG (icEEG)

#### Electrode implantation

In the 9 patients scheduled for subdural electrode implantation (RH n = 4, LH n = 5), a total of 1312 electrodes (LH n = 671; RH n = 641) were implanted (PMT Corporation; top-hat design; 3 mm diameter contact with cortex) using previously published techniques [72]. 226 electrodes (LH n = 130; RH n = 96) were excluded due to proximity to seizure onset sites, inter-ictal spikes, or 60 Hz noise. The remaining 1086 electrodes (LH n = 541, RH n = 545) were localized to cortical surface models using intra-operative photographs and an in-house recursive grid partitioning technique [73].

#### Electrode selection criteria

A combination of independent anatomical and functional criteria was used to localize all electrodes recording to early visual and face-selective inferior occipital and fusiform cortex (EVC, f-IOG, f-FG, respectively). Functional criteria for identifying face-selective electrodes were evaluated using both fMRI face-localizer and icEEG data as two independent metrics. Due to significant variability in the anatomical location of EVC, FG, and IOG electrodes across subjects, selection criteria were applied to each subject in their native brain space. This minimized the effect of inter-subject anatomical variability, and ensured that functionally homologous regions across individuals were being evaluated[62].

For each subject, anatomical criteria were used to first identify electrodes localized within the boundaries of EVC, f-IOG, and f-FG regions. EVC electrodes were localized over visual regions V1/V2/V3 [74–76], using probabilistic maps of visual topography derived from publically available retinotopic fMRI maps (Neuroscience of Attention and Perception Laboratory: http://www.princeton.edu/~napl/vtpm.htm)[74]. f-IOG electrodes were localized over the inferior occipital gyrus, lateral to the occipito-temporal sulcus and inferior to the lateral occipital sulcus [23, 31, 42]. f-FG electrodes were localized over lateral fusiform cortex, anterior to the posterior collateral sulcus, postero-medial to the occipito-temporal sulcus, and postero-lateral to the mid-fusiform sulcus [32, 77].

Functional criteria were subsequently applied to all electrodes that satisfied these initial anatomical criteria. In the EVC only those electrodes with response onset latencies less than ~100 ms, which also demonstrated similar or greater response for scrambled faces compared to face stimuli, were analyzed further [52, 78, 79]. In the f-IOG and f-FG, electrodes were evaluated for face-selectivity, using both fMRI and icEEG as independent metrics, to select only electrodes that were: 1) co-localized over face-selective fMRI clusters, and 2) significantly face-selective in a d-prime (*d*’) sensitivity analysis of the icEEG data (described below) [53, 67, 80–82]. icEEG *d*’-analysis was computed using only even-numbered trials from the visual naming tasks, to avoid circularity concerns with a subsequent analysis of face-selectivity onset latencies (performed using only odd-numbered trials).

Only electrodes meeting all criteria were considered for further analysis. We utilized this rigorous selection process to ensure that we did not over-rely on any single metric for electrode selection. One subject (subject 4, RH) but did not participate in icEEG recordings, and only underwent cortico-cortical evoked potentials (CCEPs). In this subject, EVC electrodes (n = 4) were localized over the calcarine fissure (<2cm from occipital pole), overlapping V1/V2/V3 regions identified by probabilistic visual topography maps [74, 76]. Selection of f-IOG (*n* = 2) and f-FG (*n* = 1) electrodes in this subject were guided using the anatomical criteria as well as their overlap with face-selective clusters derived from the grouped fMRI analysis in the healthy volunteer cohort.

We note here that non-invasive and intracranial neuroimaging provide substantial evidence to support the existence of multiple, distributed face-selective “areas” (or clusters/patches) in the human cortex [62, 77], and that the concept of a “single” f-FG has been recently revised to consist of two smaller clusters—a middle and posterior face-selective fusiform cortex (termed mFus-faces and pFus-faces, respectively) [30, 77]. Our goal here is to determine whether input from EVC reaches face-selective fusiform regions independently of the f-IOG. Therefore, we refer to any electrodes localized over either mFus or pFus-faces as an “f-FG” electrode, as the observation of connectivity (correlational and CCEP) between EVC and any f-FG electrode (mFus or pFus), which also precedes f-IOG and f-FG connectivity, would support this hypothesis. Thus, the grouping of electrodes from these two fusiform regions is consistent with our goal, and provides a large enough sample size to enable meaningful analysis. Care was taken to ensure that electrodes situated over pFus were not erroneously identified as f-IOG electrodes, again using sulcal and anatomical boundaries from individually parcellated cortical surface models [23].

We also acknowledge that grouping m/p-Fus electrodes precludes an investigation into the interactions of different face-selective sub-regions within the f-FG (amongst themselves and with other distributed face-selective foci). This interesting question, however, is beyond the scope of this paper, and will be considered in future analyses.

Finally, to visualize selected electrodes in a common reference space, a surface-based normalization strategy was implemented to map individual subject electrode coordinates to a standardized cortical surface (MNI N27 template brain aligned to Talairach coordinate space) [64, 65, 70, 83]. We note that due to individual anatomical variability, the visual depiction of electrodes at the group-level may not accurately reflect the location of each electrode with respect to the native cortical surface. However, the plotting of electrodes on a common brain surface in this fashion is strictly for visualization purposes. No subsequent analysis depends on the transformed location of these electrodes.

#### icEEG visual naming tasks

All subjects except for one (RH, subject 4, due to clinical constraints) participated in the icEEG language task. During icEEG recordings, subjects were asked to perform the same two visual naming tasks as they had during the functional imaging, using similar stimuli. Stimuli were displayed at eye-level on a 15” LCD screen placed at 2 feet from the patient (2000 ms on screen, jittered 3000 ms inter-stimulus interval; 500x500 pixel image size, ~10.8° x 10.8° of visual angle, with a grid overlay on 1300x800 pixel white background, ~28.1° x 17.3° of visual angle). 60 trials were presented for each condition (except animate, which had 54 trials). For each condition, images were randomly selected from our database and never repeated, so that each subject saw a unique sequence of images, with scrambled images randomly interweaved. Subjects were instructed to overtly name faces and inanimate/animate objects, and say, “scrambled” for the scrambled faces. A transistor-transistor logic pulse triggered by the stimulus presentation software (Python v2.7) at stimulus onset was recorded as a separate input during the experiments to time lock all trials.

#### icEEG processing

**Fig 1:**
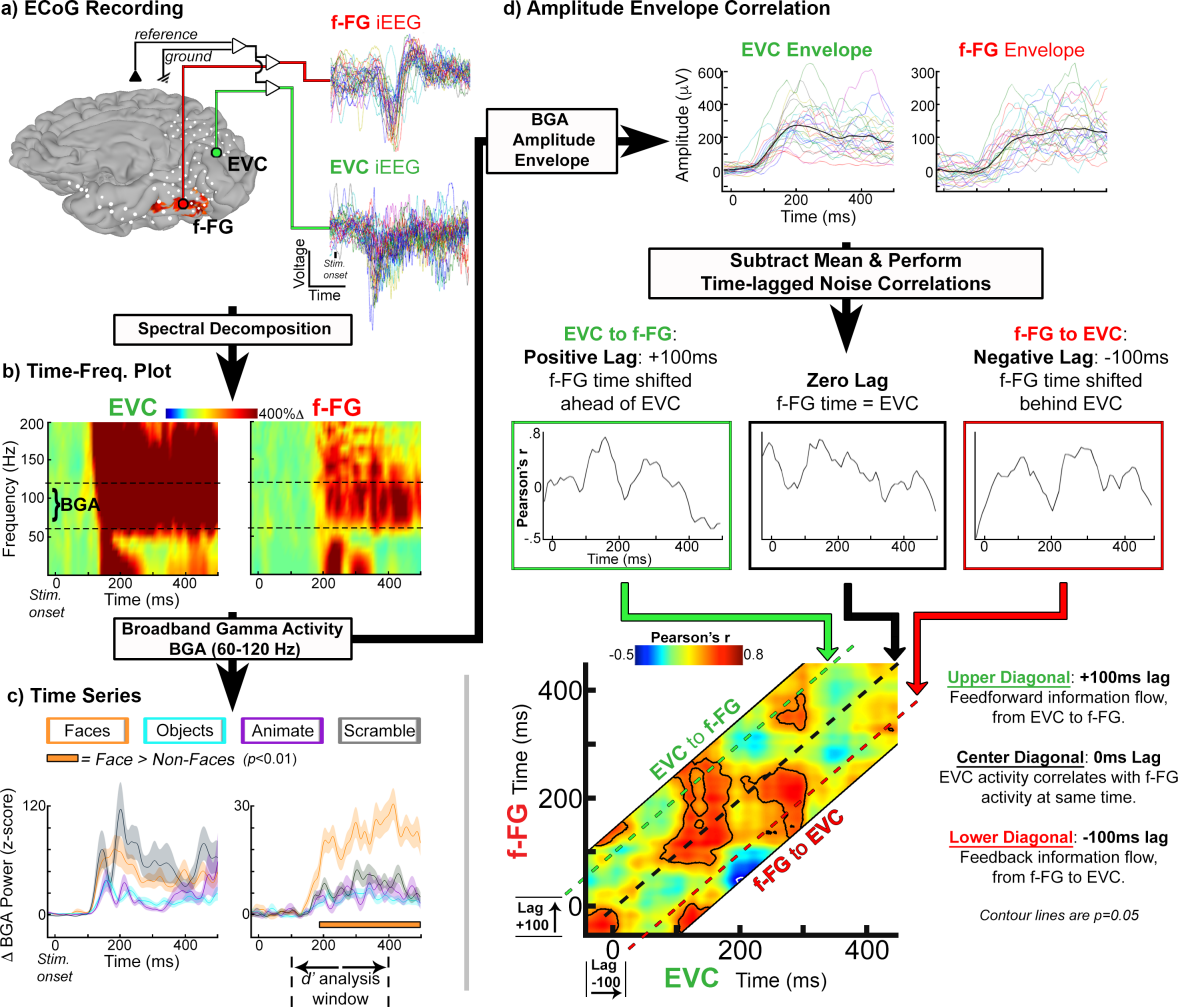
icEEG analytic methods. *(A)* Representative subject (RH, subject 1) showing analytic methods applied to the cortical activity measured using intracranial EEG (icEEG) during a face-naming task. All data are trial-aligned and from highlighted electrodes localized over early visual cortex (EVC; green) and face-selective fusiform gyrus (f-FG; red). fMRI activations depicted on the cortical surface indicate higher responses to faces than non-faces (faces > animate, inanimate, and scramble; p <0.01). fMRI data provided one of three selection criteria used to identify face-selective electrodes (via co-localization). *(B)* Time-frequency plot of percent power change (relative to pre-stimulus baseline; -700 to -200 ms; t = 0 ms, stimulus onset; face task only) following spectral decomposition of the icEEG signal. Horizontal dashed lines denote range of broadband gamma activity (BGA, 60–120 Hz) used in this study. *(C)* (*Top*) BGA profile for face (orange) and non-face control stimuli: animate (purple), inanimate (cyan), scrambled (gray) stimuli. Representative stimuli are provided in Fig 2. Shaded regions denote 1 SEM (across trials; *n* = 50–60 trials-per-condition). Horizontal orange bar denotes face-selectivity onset in f-FG (faces > non-faces; *q* <0.01, FDR corrected). Below f-FG x-axis, time window (100 to 400 ms) used for the icEEG *d*’-analysis is indicated, which independently evaluates electrode face-selectivity. To avoid circularity, odd-trials are used for selectivity onset latencies and even trials for *d’* selectivity indices. *(D)* Functional connectivity assessed using amplitude envelope correlations between electrode pairs (face task only). *(Top)* For the two electrodes here, trial-by-trial variance of instantaneous BGA envelope is obtained by subtracting the average envelope (black trace) from each trial. *(Middle)* Noise correlations performed across trials to compute connectivity between electrode pairs. To estimate information flow, zero time-lag correlations are computed (black box), and repeated for both positive (+100 ms, green box) and negative (-100 ms, red box) lag values. (*Bottom*) Temporal cross-correlograms summarize connectivity across all time-lags (-200 to +200 ms lags). Correlation coefficient values are plotted as a heat map. The black dashed line represents a 0-ms lag. Above this line, EVC activity leads f-FG (positive lag; information flow from EVC to the f-FG), while below the dashed line f-FG activity leads EVC (negative lag; information flow from f-FG-to-EVC). Contours represent significant correlations (*q* = 0.05, trial re-shuffling, 2000 resamples).

In 3 subjects, icEEG data were collected at 1000 Hz using NeuroFax software (Nihon Kohden, Tokyo, Japan) (bandwidth 0.15–300 Hz). The other 5 subjects underwent icEEG data collection at 2000 Hz (bandwidth 0.1–750 Hz) using the NeuroPort recording system (Blackrock Microsystems, Salt Lake City, UT). All electrodes with greater than 10 dB of noise in the 60 Hz band, inter-ictal epileptiform discharges, or localized to sites of seizure onset were also excluded. The remaining electrodes within each subject were referenced to a common average of all electrodes (Fig 1A) [71].

For each electrode, icEEG data were initially filtered in the broadband gamma frequency range (Fig 1B; 60–120 Hz, following removal of 60 Hz line noise and its harmonics) using a square filter with sigmoid flanks (half amplitude roll off of 1.5 Hz), and subsequently Hilbert transformed to extract the analytic amplitude envelope. To focus only on perceptual processes, all icEEG analyses were restricted to 400 ms after stimulus presentation [49, 52].

Of note, the spectral decomposition in Fig 1B was performed by bandpass filtering raw icEEG data (IIR Elliptical Filter, 30 dB sidelobe attenuation) into logarithmically spaced bands from 2 to 240 Hz. A Hilbert transform was applied to extract the analytic amplitude and derive the time course of power in each band. The percent change at each time-point was calculated by comparing power to a pre-stimulus baseline (-700 to -200 ms). This spectral decomposition was used only for the generation of the spectrogram in this figure, in order to visually depict the broadband gamma frequency range studied in this manuscript. The description of broadband gamma filtering provided in the prior paragraph is the approach utilized for the remainder of the analyses.

#### icEEG: D-Prime (d’) evaluation of face-selectivity

To quantify face-selective responses in each electrode, the d’ (d-prime) sensitivity index for faces was computed for each electrode per subject[82]. The *d*’ index is an established metric in signal detection used to determine how well a target can be discriminated from competing stimuli [53, 67, 80, 81]. For each electrode, and for each task condition, mean BGA in the 100-400ms interval after stimulus onset was normalized by across-trial standard deviation (even trials only) [67, 81, 82]. The d’ index was calculated as the difference between the standardized BGA for faces against all other non-face categories (Eq 1):

$$d'=\frac{\mu_{j}-\frac{1}{N}\sum_{i}^{N}\mu_{i}}{\sqrt{\frac{1}{2}\left(\sigma_{j}^{2}+\frac{1}{N}\sum_{i}^{N}\sigma_{i}^{2}\right)}};i\neq j$$ (1)

where *u*_j_ is the mean BGA to the faces; *o*_j_ is across-trial standard deviation of BGA activity to faces; and *u*_i_ and *o*_i_ denote the same for the other conditions. *N* is equal to 3. Significance thresholds were determined through permutation testing[82]. For each electrode per subject, a null distribution was generated by randomly shuffling category labels across all trials and recomputing the d’ index 10,000 times. The p-value for each electrode was determined as the fraction of shuffled d’ indices that were greater than the actual d’ index [81, 82].

We note again that the icEEG *d*’-analysis was computed using only even trials. This was done to avoid circularity concerns with our subsequent analysis of face-selectivity onset latencies, which were performed using only odd-trials in the icEEG data.

#### icEEG: Time-series evaluation of face-selectivity onset latency

To evaluate face-selectivity onset latencies in f-IOG and f-FG electrodes, time-series representations of mean BGA (across trial; odd-trials only) were computed at each electrode per subject for face, animate, inanimate, and scramble face stimuli (Fig 1C; time windows extend from -50 to 400 ms following stimulus onset; stimulus onset at *t* = 0 ms). For each electrode, per task condition, BGA analytic amplitude was baseline normalized (Z-scored) with respect to BGA power in a pre-stimulus window (-700 to -200 ms).

Onset of face-selectivity in a given electrode was defined as the first time point at which a significant face vs. non-face contrast was observed, which then remained significant for >100 ms. This was determined using paired two-way t-tests performed at each time point (faces > inanimate/animate/scrambled faces*; q <* 0.01; corrected for multiple time-point comparisons using the false discovery rate procedure) [84].

The millisecond temporal resolution afforded by icEEG allows for precise latency estimates [76]. To determine whether onset latencies were significantly different between f-IOG and f-FG electrodes, paired contrasts were computed between all f-IOG-vs-f-FG electrode combinations for each subject, and then combined across all subjects. For example: if subject A had 2 IOG and 3 FG electrodes, and subject B had 1 IOG and 4 FG electrodes, then subject A will have a total of 6 inter-areal comparisons and subject B will have a total of 4 inter-areal comparisons. The final statistic will then be computed over all 10 difference values (6 from subject A + 4 from subject B) using the two-sided Wilcoxon sign-rank test to test the null hypothesis that the true difference is 0.

#### icEEG: Amplitude envelope correlation evaluation of functional connectivity

A full description of network dynamics depends on both the patterns of cortical activation and the functional connectivity that underpins them [47]. However, traditional connectivity analyses that utilize phase relationships to study neural synchronization [85] are poorly suited to the asynchronous nature of high-frequency BGA [58, 86]. We therefore sought to categorize cortical interactions using amplitude envelope correlations (Fig 1E), which circumvents this issue by computing coupling between BGA power envelopes, independent of the phase [58, 59, 87, 88], and thus provides an estimate of the co-variance between two regions actively engaged in a task. Importantly, amplitude envelope correlations can be performed at varying time lags [87], enabling directionality connectivity estimates between more distant cortical regions that may not share instantaneous (i.e. zero-lag) correlations.

BGA amplitude envelope of each trial was smoothed using a moving average filter (50 ms) (Fig 1E; analysis time window is from -50 to 400 ms following stimulus onset; stimulus onset is at *t* = 0 ms). The average across trials was then subtracted from the amplitude envelope to obtain trial-by-trial variance for each electrode. Noise correlations between pairs of electrodes were computed using Pearson’s correlation of the variance at each time point across trials. The low signal amplitude (2–5 microvolts in the 60–120 Hz band) in the gamma frequency range, together with the use of noise correlations, ensures that signal overlap and therefore spurious correlations between channels are unlikely [58, 86, 87].

Given that connectivity between distant cortical regions may not be completely represented by instantaneous correlations (i.e. at zero time lag), we also calculated trial-by-trial correlations at more extended time lags. For each electrode pair, we lagged the time series on one channel prior to computing noise correlations, with a maximum lag of 150 ms. In this manner, amplitude envelope correlations can estimate the directionality of information flow by correlating activity in one region against activity in another region at an earlier or later point in time [87]. Temporal cross-correlograms were used to summarize noise correlations calculated across all time lags between regions (Fig 1E).

To achieve grouped estimates of connectivity, the electrodes localized in each region per individual (EVC, f-IOG, f-FG) were used to generate a list of possible pairs between these regions. Amplitude envelope correlations were first computed at the individual level by averaging correlograms across all respective electrode pairs within a subject. Individual correlograms were then transformed into a Fisher’s z, averaged across subjects, and assessed for significance.

Significance for connectivity estimates was calculated against shuffled data (*q* = 0.05, individual level; *q* = 0.01, group level; corrected for multiple comparisons using the false detection rate method; trial re-shuffling performed using 2000 resamples using MATLAB Parallel Computing Toolbox ver 6.1)[88]. Shuffled data were utilized to ensure that patterns of connectivity observed were not due to event-related coupling from signal changes common to all trials (e.g. event related potentials). To achieve this, noise correlations were re-computed for each individual electrode pair by shuffling the trial order (but not the temporal information, e.g. time points) in the second electrode with respect to the first. Trial randomization was performed with replacement, such that a given trial could be repeatedly drawn, or not drawn at all, with equal probability.

In a bootstrap procedure, shuffling was repeated for 2000 randomizations to generate a null distribution for the coupling value between a given pair of electrodes[88]. Individual-level *p*-values were determined using the fraction of shuffled correlation coefficients that exceeded the actual correlation value[81, 82]. At the group level, each shuffled dataset (*n* = 2000) was Fisher z-transformed and averaged across subjects in the same fashion as the original, trial-congruent analysis. Group-level *p-*values were determined using the fraction of averaged shuffled Fisher transformed correlations that exceed the actual grouped correlation value. All *p*-values (individual and grouped-level) were corrected for multiple-comparisons (across time-points and lags) using the false-detection rate method (*q* = 0.05 individual; *q* = 0.01 group).

#### Cortico-cortical evoked potentials (CCEPs)

Given that individual subject electrode placement is both sparse and variable, connectivity between EVC and the f-IOG and f-FG might reflect interactions from unrecorded neural substrates (i.e. hidden/common-source correlations). We therefore also utilized cortico-cortical evoked potentials (CCEPs) to evaluate electrophysiological connectivity between these regions in a task-independent fashion [60, 89–91]. CCEPs offer a direct means of mapping signal propagation latencies between structurally coupled cortical networks, using repeated delivery of single- pulse electrical stimulation at a single pair of subdural electrodes [89, 92]. Another advantage of CCEP mapping is the ability to evaluate reciprocity of signal propagation between two stimulation sites, which can suggest whether information flows in a unidirectional or bidirectional fashion[93].

CCEPs were derived using bipolar stimulation of selected cortical regions (10 mA, 500 micro-second pulse width at 1 Hz for 50s) with a Grass Stimulator (Grass Technologies, West Warwick, RI USA) [90]. Concurrent icEEG was recorded at 1 kHz using NeuroFax software (Nihon Kohden). A subgroup of electrodes, located more than 2 cm from the stimulation site and with minimal stimulus artifact was used to generate an average reference. icEEG data were exported to Matlab, and time locked to the beginning of each stimulus. Noisy trials containing inter-ictal epileptiform discharges or artifacts were excluded from further analysis. A high pass filter (10th order Chebyshev, 1 Hz cutoff, 30 dB side lobe attenuation) was applied to each channel to minimize the effects of voltage drift. Epochs were then averaged to derive the CCEP at each recording electrode. Positive and negative deflections in the averaged CCEP response at each electrode were identified using an automated peak detection algorithm (in-house software) [90]. Data within the first 10 ms were excluded to eliminate stimulation artifact. The first voltage deflection following the stimulus artifact was defined as an N1 response (irrespective of polarity) [60, 89]. The N1 response latency was calculated as the difference between the timing of the stimulus artifact and this first voltage deflection. Only voltage deflections within 40 ms of stimulus artifact were classified as N1 responses to minimize the influence of indirect connections. Channels with N1 peak amplitudes >1000 mV were excluded, as they likely reflected non-biological electrical transmission.

N1 voltage deflections are thought to result from a two-stage physiological process induced at the stimulation site and the evoked response at the recording site [89]. During stimulation, induced changes are believed to result from the direct depolarization of superficial dendritic trees of local pyramidal cells. While stimulation has also been demonstrated to induce depolarization in adjacent inhibitory interneurons, as well as in long-range axons traversing within the region of stimulation, animal studies suggest that CCEPs at the recording site result from the depolarization of middle and deep pyramidal cells [89, 94]. This is supported by laminar current source density analyses demonstrating pyramidal cell depolarization following transient sensory stimulation, with early (10 – 30ms) cortical excitation patterns strongly resembling the N1[89, 95, 96]. The latency of the N1 peak (< = ~40 ms) suggests that feed-forward signal propagation from stimulation sites occurs via oligo- or polysynaptic pathways (given that induced responses via monosynaptic connections are expected to occur following a 4–8 ms delay[89]). In sum, these studies suggest that the N1 reflects neuronal activation from direct axonal projections. The relationship between the N1 and underlying structural connections has been reaffirmed by prior work in our lab, correlating N1 response properties (e.g. strength and latency) with structural connectivity measures using DTI[90].

## Results

Subdural electrodes, localized to the EVC, f-IOG and f-FG were identified in all subjects using anatomical and independent functional criteria. In the right hemisphere, 34 electrodes were localized over EVC (*n* = 16), f-IOG (*n* = 8), and f-FG (*n* = 10). In the left hemisphere, 39 electrodes were localized over EVC (*n* = 16), f-IOG (*n* = 8), and f-FG (*n* = 15). One subject (subject 4) with right hemispheric coverage did not undergo icEEG recordings, and is excluded from the time-series and functional connectivity analyses.

IcEEG analyses were conducted using broadband gamma power (BGA; 60–120 Hz) as a key electrophysiological measure of cortical activity (Fig 1). BGA was selected given that it has demonstrated to provide precise estimates of task-specific cortical activity [49, 52, 78, 97] as well as the strongest correlation with the BOLD fMRI signal used in non-invasive neuroimaging studies[63, 67, 68, 98].

### Latency differences in f-IOG and f-FG face-selectivity

**Fig 2:**
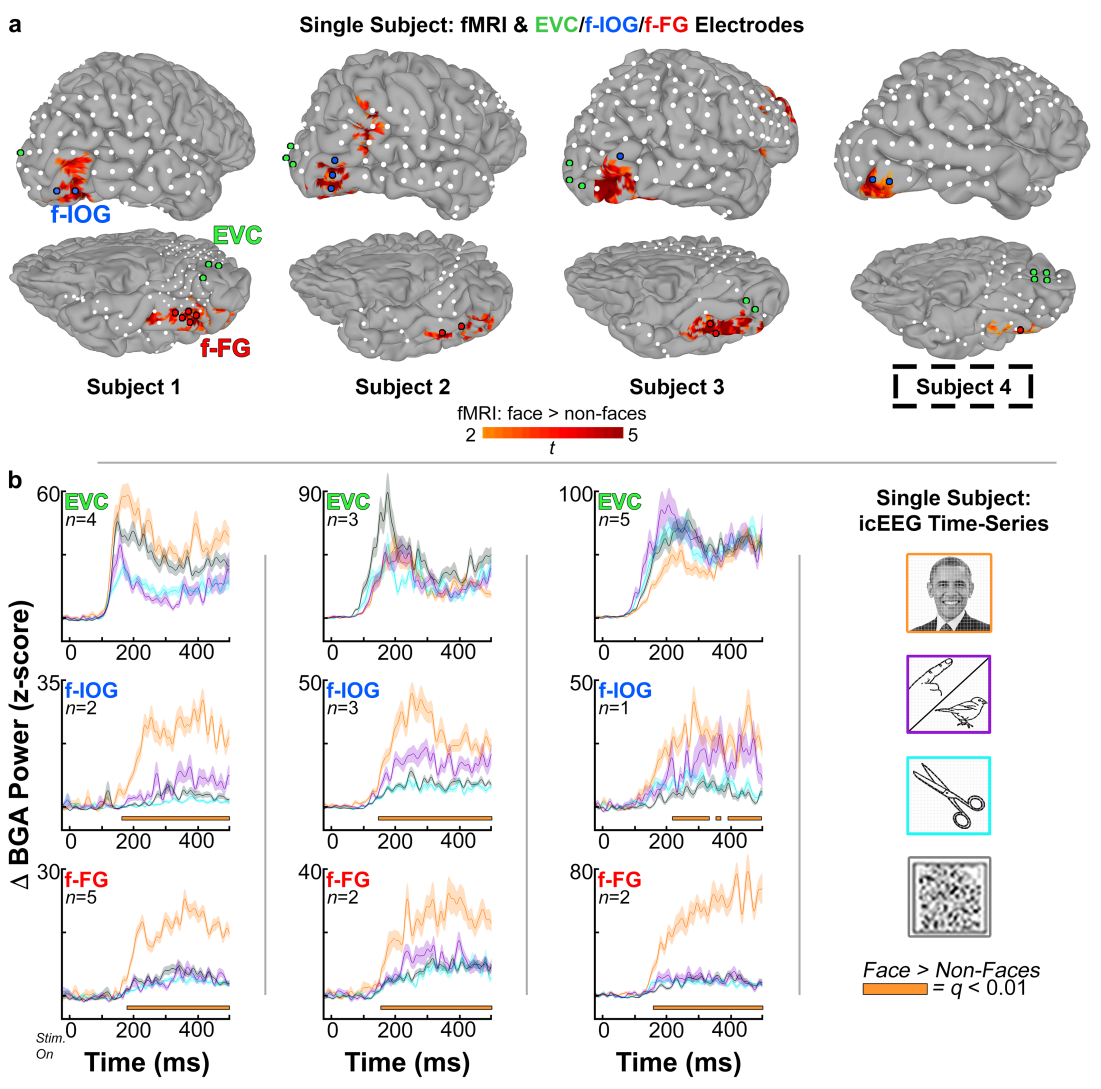
Right hemisphere subject electrodes, fMRI, and icEEG time-series. *(A)* Cortical surface models and subdural electrodes are shown for the subjects with right hemispheric coverage in all three regions of interest. Highlighted electrodes (colored spheres) met all anatomical and functional criteria for early visual cortex (EVC, green), and face-selective inferior occipital gyrus (f-IOG, blue) and fusiform gyrus (f-FG, red). Subject-specific fMRI activations, also depicted on cortical surfaces (subjects 1–3), indicate higher responses to faces than non-faces (faces > animate, inanimate, and scramble; *p* <0.01). fMRI data provided one of three selection criteria used to identify face-selective electrodes (via co-localization). Subject 4 was unable to participate in fMRI recordings (dashed black boxes). fMRI activations depicted in this last case are derived from grouped analysis in 18 healthy volunteers, co-registered to the subject’s own cortical model using a surface-based normalization technique. *(B)* icEEG time-series representations of normalized (z-scored) mean broadband gamma power changes (BGA; 60–120 Hz; -50 to 700 ms after stimulus onset; stim. on at *t* = 0 ms), with respect to pre-stimulus baseline (-700 to -200 ms), for EVC, f-IOG, and f-FG electrodes identified in each subject. Time-series traces are color-coded to respective stimulus category–faces (orange) vs. animate (purple) vs. inanimate (cyan) vs. scramble (gray) stimuli. Shading denotes 1 SEM (across electrodes/region/subject; *n* value). Horizontal orange bars below each trace represent onset of BGA face-selectivity used in f-IOG and f-FG latency difference contrasts (face > non-face stimuli; *q* < 0.01, FDR corrected for time-points; computed using odd-trials). Subject 4 did not undergo icEEG recordings. Note: Line drawings of non-face stimuli (Figs 1–4) were adapted with permission from: Snodgrass J.G. and Vanderwart M. "A standardized set of 260 pictures: Norms for name agreement, image agreement, familiarity, and visual complexity.” Journal of Experimental Psychology: Human Learning and Memory, Vol 6(2), 1980, 174–215, APA. The official portrait of President Barack Obama by Pete Souza (Figs 1–4), obtained from the White House website (https://www.whitehouse.gov/sites/whitehouse.gov/files/images/Administration/People/president_official_portrait_hires.jpg), is licensed under CC BY 3.0 US (http://creativecommons.org/licenses/by/3.0/us/) as pursuant to White House copyright policy (https://www.whitehouse.gov/copyright/). Original images have been converted to gray-scale and overlaid with grid.

**Fig 3:**
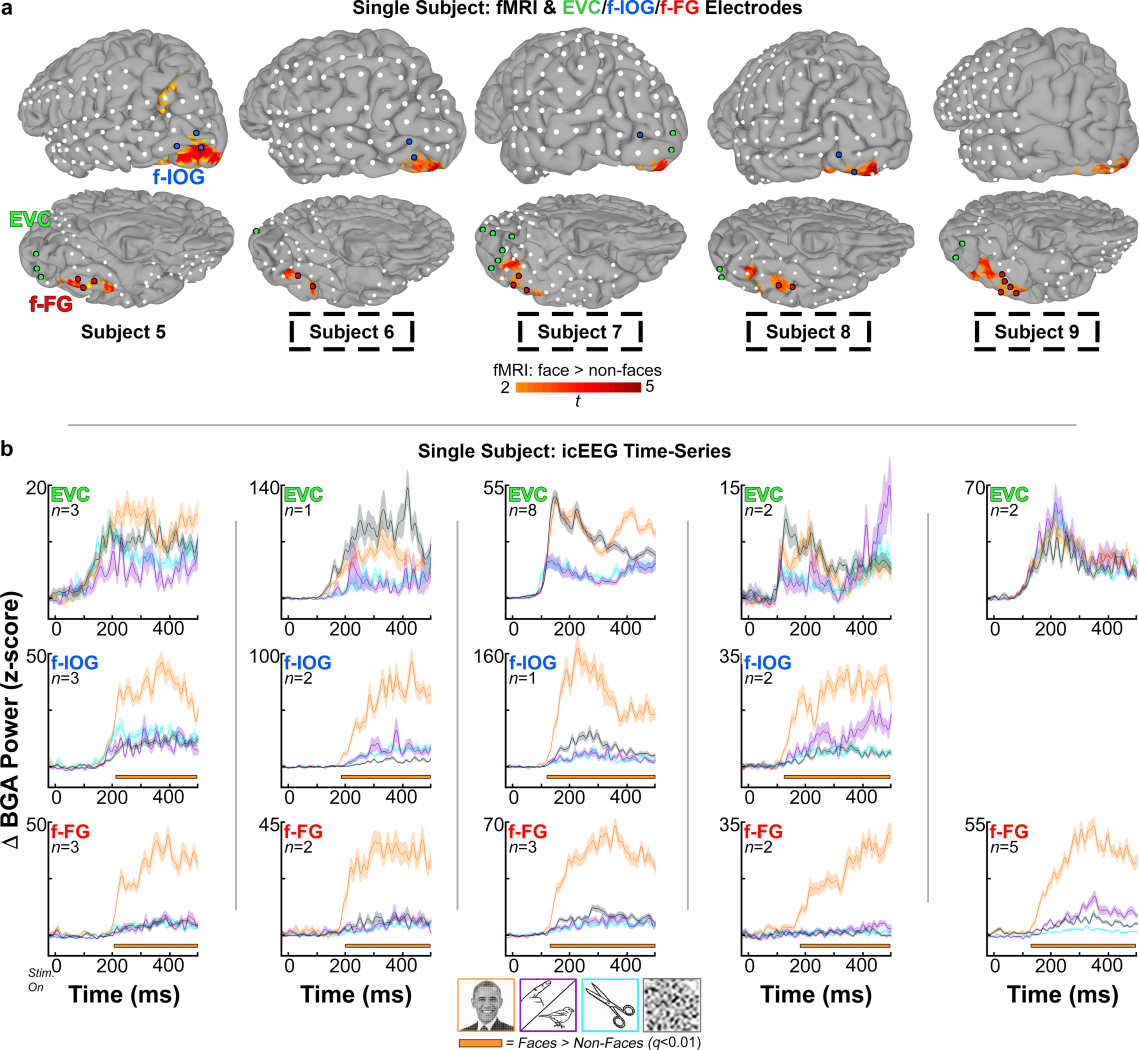
Left hemisphere subject electrodes, fMRI, and icEEG time-series. A) Cortical surface models and subdural electrodes are shown for the subjects with left hemispheric coverage in all three regions of interest. Highlighted electrodes (colored spheres) met all anatomical and functional criteria for early visual cortex (EVC, green), and face-selective inferior occipital gyrus (f-IOG, blue) and fusiform gyrus (f-FG, red). Subject-specific fMRI activations, also depicted on cortical surfaces (subject 5), indicate higher responses to faces than non-faces (faces > animate, inanimate, and scramble; *p* <0.01). fMRI data provided one of three selection criteria used to identify face-selective electrodes (via co-localization). For subjects that did not participate in fMRI recordings (dashed black boxes; subjects 6–9), the fMRI activations depicted derive from a grouped analysis in 18 healthy volunteers, which was co-registered to the subject’s own cortical model using a surface-based normalization technique. B) icEEG time-series representations of normalized (z-scored) mean broadband gamma power changes (BGA; 60–120 Hz; -50 to 700 ms after stimulus onset; stim. on at *t* = 0 ms), with respect to pre-stimulus baseline (-700 to -200 ms), for EVC, f-IOG, and f-FG electrodes identified in each subject. Time-series traces are color-coded to respective stimulus category–faces (orange) vs. animate (purple) vs. inanimate (cyan) vs. scramble (gray) stimuli. Shading denotes 1 SEM (across electrodes/region/subject; *n* value). Horizontal orange bars below each trace represent onset of BGA face-selectivity used in f-IOG and f-FG latency difference contrasts (face > non-face stimuli; *q* < 0.01, FDR corrected for time-points; computed using odd-trials).

EVC, f-IOG, and f-FG electrodes identified by independent anatomical and functional criteria are depicted on subject-specific cortical subject models in Figs 2A and 3A (colored spheres; right and left hemisphere cohort, respectively), and are visualized in relation to fMRI-localized face-selective activations (one of the functional selection criteria).

In the right hemisphere (Fig 2A): Subject 1 had 4 electrodes localized over EVC (*d’* range: 0.19, 1.18), 2 over f-IOG (*d*’ range: 1.82, 2.88), and 5 over f-FG (*d*’ range: 1.61, 2.35). Subject 2 had 3 EVC (*d’* range: -0.70, 0.13), 3 f-IOG (*d*’ range: 0.62, 3.23), and 2 f-FG electrodes (*d*’ range: 0.75, 2.64). Subject 3 had 5 EVC (*d’* range: -1.12, 0.14), 1 f-IOG (*d*’ index: 1.58), and 2 f-FG (*d*’ range: 2.08, 2.68) electrodes. Subject 4 had 4 EVC, 2 f-IOG, and 1 f-FG electrodes (note: subject 4 did participate in icEEG recordings, and did not contribute to icEEG latency or functional connectivity analyses). All *d’*-estimates for f-IOG and f-FG were significant at an individual-level *p* = 0.05.

In the left hemisphere (Fig 3A), subject 5 had 3 EVC (*d’* range: 0.39, 0.94), 3 f-IOG (*d*’ range: 0.84, 2.19), and 3 f-FG electrodes (*d*’ range: 1.59, 3.34). Subject 6 had 1 EVC (*d’* index: 0.029), 2 f-IOG (*d*’ range: 1.73, 2.93), and 2 f-FG (*d*’ range: 2.23, 2.41) electrodes. Subject 7 had 8 EVC (*d’* range: -0.39, 1.24), 1 f-IOG (*d*’ index: 1.63), and 3 f-FG (*d*’ range: 1.05, 2.25) electrodes. Subject 8 had 2 EVC (*d’* range: -0.39, 0.49), 2 f-IOG (*d*’ range: 0.89, 1.63), and 2 f-FG (*d*’ range: 1.19, 1.56) electrodes. Subject 9 had 2 EVC (*d’* range: -0.03, 0.52), 0 f-IOG, and 5 f-FG (*d*’ range: 1.87, 4.30) electrodes. All *d’*-estimates for f-IOG and f-FG were significant at an individual-level *p* = 0.05. Subjects 6–9 (LH) were unable to participate in fMRI visual naming tasks. The fMRI data depicted on their cortical surface models were derived from a surface-based, mixed-effects multilevel grouped analysis from 18 healthy subjects. Differences in location and appearance of healthy grouped fMRI results on the cortical surfaces of subjects 6–9 are a result of the non-linear transformations used to align cortical surface models across subjects, and are a testament to the enormous variability on cortical folding patterns in humans.

icEEG time-series representations of normalized (z-scored) mean broadband gamma power changes (BGA; 60–120 Hz; -50 to 700 ms after stimulus onset; stim. on at *t* = 0 ms), with respect to pre-stimulus baseline (-700 to -200 ms), for EVC, f-IOG, and f-FG electrodes identified in each subject. Time-series traces are color-coded to respective stimulus category–faces (orange) vs. animate (purple) vs. inanimate (cyan) vs. scramble (gray) stimuli. Shading denotes 1 SEM (across electrodes/region/subject; *n* value). Horizontal orange bars below each trace represent onset of BGA face-selectivity used in f-IOG and f-FG latency difference contrasts (face > non-face stimuli; *q* < 0.01, FDR corrected for time-points; computed using odd-trials).

Across all LH and RH subjects, f-FG electrodes identified by anatomical and icEEG (*d*-prime) functional criteria were reliably localized over face-selective FG clusters identified by fMRI. This tight coupling in f-FG was even consistent with LH subjects 6–9 using the healthy subject grouped fMRI data. This finding is consistent with prior reports demonstrating a strong correspondence between the BOLD fMRI signal and high-frequency icEEG neural activity (e.g. broadband gamma activity), especially for face-selective clusters in the fusiform gyrus[63, 67, 68, 78, 98–100]. However, while all face-selective f-FG electrodes were localized over face-selective fMRI clusters, the converse was not necessarily true. For the 4 LH subjects (6–9) using the healthy subject fMRI data, more posterior f-FG face-selective fMRI clusters were observed to be localized under electrodes not exhibiting face-selectivity in the *d*-prime and/or time-series analyses [23, 26, 30]. Correspondence between fMRI and icEEG was also less consistent for f-IOG electrodes. While f-IOG electrodes in subjects using their own fMRI data did demonstrate strong correspondence (RH subjects 1–3; LH subject 5), for the 4 LH subjects (6–9) using the healthy subject fMRI data, only 2 of 5 f-IOG electrodes co-localized with face-selective fMRI activity. We note that correspondence errors between fMRI and icEEG face-selectivity, in the 4 LH subjects using group-transformed data, may be due to inter-subject co-registration errors with the healthy subject fMRI data. Alternatively, they may be due to fMRI artifact contamination resulting from the presence of the transverse venous sinus (often most severe in the left hemisphere)[101]. A final important consideration is the possibility that these correspondence errors may be magnified by inter-subject variability from the healthy subjects used to derive the grouped fMRI results, who indeed demonstrated a greater degree of variability in their peak values for the left hemispheric face-selectivity clusters than in the right (S3 Fig). More importantly, these errors demonstrate the value of using multiple independent metrics to identify face-selective electrodes.

Next, we computed face-selectivity onset latencies in each f-IOG and f-FG electrode per subject. Onset latencies were computed by contrasting normalized (z-scored) BGA for faces vs. high- and low-level non-face control stimuli. In the right hemisphere (Fig 2B), mean face-selectivity onset latencies (computed across f-IOG and f-FG electrodes separately) were [f-IOG vs. f-FG ms]: [162 vs. 179 ms] in subject 1, [146 vs. 150 ms] in subject 2, and [218 vs. 157 ms] for subject 3. In the left hemisphere (Fig 3B), mean selectivity onset latencies in f-IOG and f-FG electrodes were [f-IOG vs. f-FG ms]: [211 vs. 207 ms] in subject 5, [186 vs. 201 ms] in subject 6, [119 vs. 132 ms] for subject 7, [125 vs. 185 ms] for subject 8, and [n/a vs. 121 ms] for subject 9. Significant face vs. non-face contrasts were determined at an individual corrected alpha level of *q* = 0.01. Overall, mean f-IOG and f-FG onset latencies were consistent with timing estimates from prior literature on the temporal markers of higher-level visual face processing (~120 to 200 ms; e.g. M170/N170/N200) [49, 55, 56, 68, 102, 103].

Next, to compare selectivity onset latencies between f-IOG and f-FG electrodes, a difference value (i.e. onset delay) was computed for each unique f-IOG–f-FG electrode pair per subject. By using difference values, inter-subject variability in onset latency could be taken into account. These difference values were negative if f-IOG selectivity onset preceded f-FG, and positive if f-FG precedes f-IOG.

In the right hemisphere, for subject 1, the median (first quartile, third quartile) onset latency difference value for the 10 paired-electrode contrasts (2 f-IOG x 5 f-FG) was -31 ms (-43.5, 19 ms). In subject 2, for the 6 paired-electrode contrasts (3 f-IOG x 2 f-FG), the median latency difference value was 11.5 ms (-6, 29 ms). In subject 3, for the 2 paired-electrode contrasts (1 f-IOG x 2 f-FG), the median latency difference values were 54 ms and 6 ms. Of note, 1 f-FG electrode from subject 1, as well as 1 f-IOG and 1 f-FG electrode from subject 2 were excluded from this portion of the analysis because their onset latencies did not survive statistical criteria for the selectivity onset calculations (>100 ms significant difference at a FDR corrected *q* = *0*.*01*), resulting in a total of 8 and 2 paired-electrode contrasts for each of these subjects, respectively. Overall, across all subjects in the right hemisphere, selectivity onset latencies were not significantly different between f-IOG and f-FG electrodes, with a median difference value of 0 ms (-42, 24.5 ms) (*p* = 0.63, two-sided, non-parametric Wilcoxon sign-rank test). Latency difference values for the right hemisphere are further summarized by the frequency distribution presented in Table 1.

**Table 1:** Frequency distribution of differences in f-IOG vs. f-FG selectivity onset latencies, right hemisphere.

Range: Latency Differences (ms)	Frequency	Relative Frequency	Cumulative Frequency
-64, -37	4	0.333	4
-37, -10	1	0.083	5
-10, 17	2	0.167	7
17, 44	5	0.417	12

Negative latency difference values indicate f-IOG selectivity onset precedes f-FG. Positive latency difference values indicate f-FG selectivity onset precedes f-IOG.

In the left hemisphere, for subject 5, the median (first quartile, third quartile) onset latency difference value for the 9 paired-electrode contrasts (3 f-IOG x 3 f-FG) was 2 ms (-55, 19 ms). In subject 6, for the 4 paired-electrode contrasts (2 f-IOG x 2 f-FG), the median latency difference value was -11 ms (-13, -9 ms). In subject 7, for the 3 paired-electrode contrasts (1 f-IOG x 3 f-FG), the median latency difference value was -37 ms (-57.25, -19.75 ms). In subject 8, for the 4 paired-electrode contrasts (2 f-IOG x 2 f-FG), the median latency difference value was -38 ms (-59.5, -16.5 ms). Subject 9 did not have any f-IOG electrode coverage, and was not included in the latency difference comparisons. Of note, 1 f-IOG electrode from subject 5 and 1 f-IOG electrode from subject 6 were excluded from this portion of the analysis as their onset latencies did not survive statistical criteria for the selectivity onset calculations (>100 ms significant difference at a FDR corrected *q* = *0*.*01*), resulting in a total of 6 and 2 paired-electrode contrasts for each of these subjects, respectively. Overall, across 4/5 subjects in the left hemisphere, selectivity onset latencies in f-IOG electrodes significantly preceded the f-FG, with a median difference value of -14 ms (-50.75, 3 ms) (*p* = 0.0197, two-sided, non-parametric Wilcoxon sign-rank test). Latency difference values for the left hemisphere are further summarized by the frequency distribution presented in Table 2.

**Table 2:** Frequency distribution of differences in f-IOG vs. f-FG selectivity onset latencies, left hemisphere.

Range: Latency Differences (ms)	Frequency	Relative Frequency	Cumulative Frequency
-82, -61	4	0.267	4
-61, -40	1	0.066	5
-40, -19	3	0.200	8
-19, 2	4	0.267	12
2, 23	3	0.200	15

Negative latency difference values indicate f-IOG selectivity onset precedes f-FG. Positive latency difference values indicate f-FG selectivity onset precedes f-IOG.

#### Functional connectivity through amplitude envelope correlations

We use the symbol “⇒” to indicate the direction of positive unidirectional correlations, while bidirectional correlations are represented by the “⇔” symbol. All connectivity measures were tested at a corrected significance level of *q* = 0.01 with false-detection rate correction for multiple time-point and lag comparisons at the group level. Results observed at the group level were also notable in analyses performed between individual subject electrode pairs. Correlational analyses were evaluated within the same time window (<400 ms; stimulus onset at *t* = 0 ms) as our other icEEG analyses. This time window is also centered around the estimated face-selectivity onset latencies from our icEEG time-series analysis (~120 to 200 ms).

**Fig 4:**
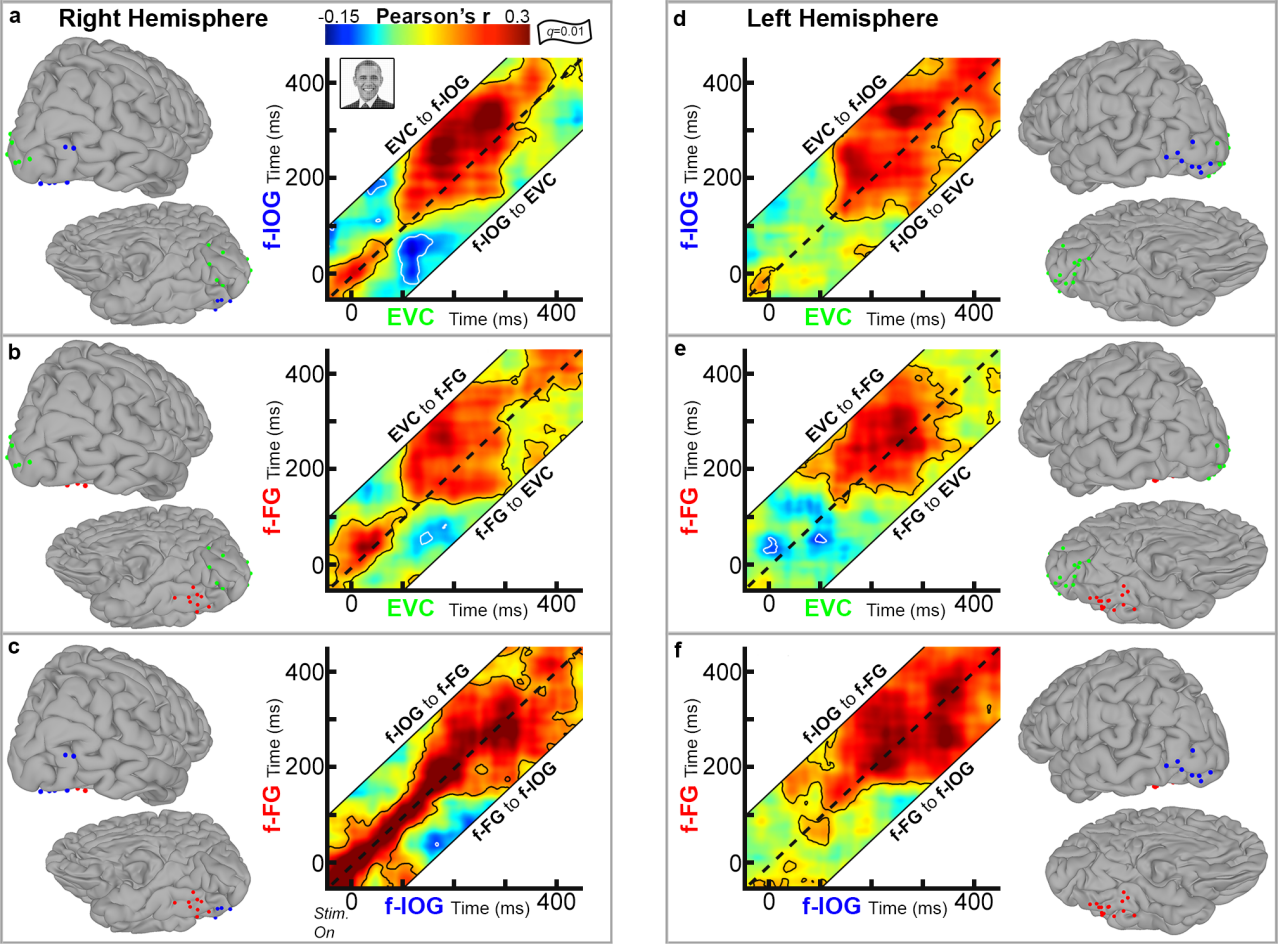
Functional connectivity during face perception. *(A)* Group temporal cross correlograms of right hemisphere EVC-f-IOG connectivity, computed by averaging individual amplitude envelope correlations (*n* = 3 subjects, contours denote significant connectivity, *q* = 0.01, FDR corrected) for face stimuli only. Amplitude envelope correlations are measured across lag ranges of -150 to +150 ms. The black dashed diagonal line represents a lag of 0 ms. Above the dashed line activity in EVC activity leads f-IOG (information flow from EVC to the f-IOG), while below the dashed line f-IOG activity leads EVC (information flow from f-IOG to EVC). *(B)* Connectivity between EVC and the f-FG, right hemisphere (*n* = 3 subjects). *(C)* Connectivity between f-IOG and f-FG, right hemisphere (*n* = 3 subjects). *(D)* Connectivity between EVC and the f-IOG, left hemisphere (*n* = 4 subjects). *(E)* Connectivity between EVC and the f-FG, left hemisphere (*n* = 5 subjects). *(F)* Connectivity between f-IOG and the f-FG, left hemisphere (*n* = 4 subjects).

In the right hemisphere (Fig 4A–4C), significant positive correlations were present between all three regions at pre-stimulus baseline (*t* < 0 ms). Between EVC and f-IOG, significant baseline correlations ended ~75ms after stimulus onset. At ~100ms, significant feed-forward EVC⇒f-IOG connectivity re-emerged, which continued until ~400 ms (Fig 4A). Between EVC and f-FG, significant baseline correlations continued until ~100 ms after stimulus onset. At ~100 ms, significant feed-forward EVC⇒f-FG connectivity then emerged, which became briefly bidirectional before ending by ~400ms (Fig 4B). Subsequent bidirectional EVC⇔f-FG correlations were noted beginning after ~400 ms, which continued beyond the current analysis window. Between f-IOG and f-FG, strong positive correlations observed at baseline continued until ~150 ms after stimulus onset (Fig 4C). At ~150ms, the magnitude in correlation coefficient decreased, as significant f-IOG⇔f-FG connectivity became broadly bidirectional until ~400ms, after which connectivity continued in a feed-forward fashion (Fig 4C). Importantly, the task-dependent changes observed between f-IOG and f-FG (~150ms) were bi-directional in nature (not solely feed-forward), and were preceded by the onset of significant feed-forward EVC⇒f-FG connectivity (S1 Fig).

In the left hemisphere (Fig 4D–4F), significant baseline connectivity was observed only between EVC and f-IOG, which ended following stimulus onset. At ~110ms, positive, bidirectional EVC⇔f-IOG connectivity re-emerged and continued until ~400ms, at which point it extended beyond the analysis window in a feed-forward fashion (Fig 4D). Between EVC and f-FG, significant positive correlations emerged at ~100ms after stimulus onset, and continued as feedforward EVC⇒f-FG connectivity until ~400ms (Fig 4E). Between the f-IOG and f-FG, positive, feedforward f-IOG⇒f-FG emerged by ~50ms, which then became bidirectional by ~100ms and continued through the remainder of the analysis window (Fig 4F). Notably, for the left hemisphere, f-IOG⇒f-FG connectivity was first to emerge following stimulus onset, preceding both EVC⇒f-FG (~100ms) and EVC⇒f-IOG connectivity (~110ms) (S1 Fig).

We note that the use of a moving averaging to smooth BGA envelope time series prior to individual noise correlation analysis could give rise to false correlations. To confirm that this was not the case, the amplitude envelope correlations were reanalyzed using 5ms, non-overlapping intervals. Critically, non-smoothed correlational analyses remained consistent with our original results in valence, directionality, and timing of connectivity onset (S2 Fig), confirming that our results were not a spurious result due to smoothing.

#### Cortico-cortical evoked potentials (CCEPs)

Stimulation was performed at both EVC and f-FG electrodes (10 mA, 500 micro-second pulse-width at 1Hz for 50s; n = 50 pulses; See Experimental Procedures). During stimulation, voltage deflections evoked within the first 10 to 40 ms were analyzed in electrodes over non-stimulated regions of interest. This early evoked response, termed the N1 response, is believed to reflect local excitations resulting from direct axonal projections from the stimulation site [89, 90].

To estimate the relative path of signal propagation from the stimulation site, we compared N1 latencies between electrodes recording from non-stimulated regions. If CCEPs were not significantly different in two distinct recording zones (e.g. f-IOG and f-FG), it suggests that neural propagation from the stimulation site (e.g. the EVC) reaches both regions through parallel axonal projections. Alternatively, if CCEPs in one region (e.g. the f-IOG) were consistently observed prior to the other (f-FG), it implies a more serial propagation of electrical activity between these regions.

**Fig 5:**
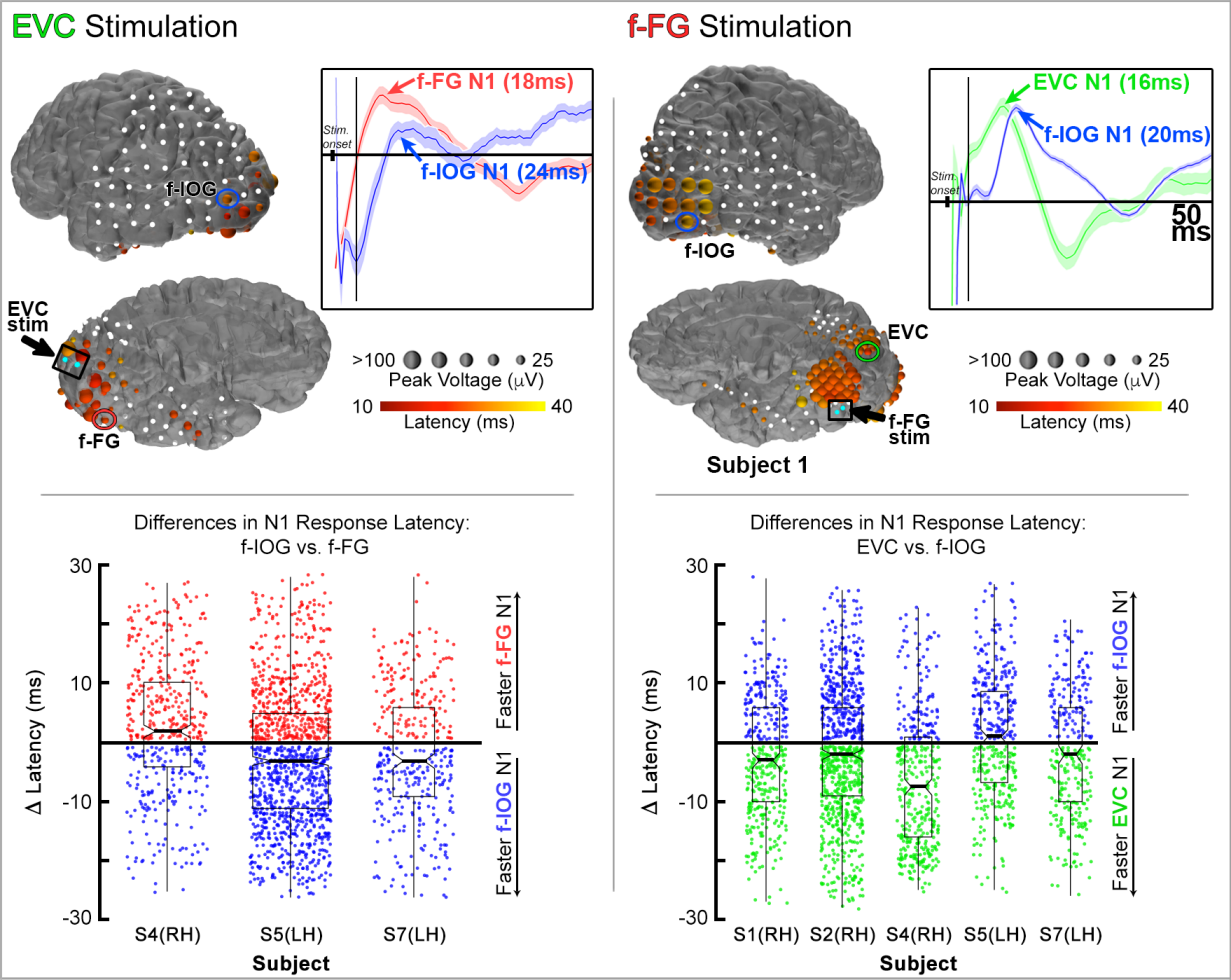
Cortico-cortical connections of the face-network. (Top) Cortico-cortical evoked potentials (CCEPs) visualized on subject cortical surface models during stimulation at a pair of early visual cortex (EVC; left) and face-selective fusiform gyrus (f-FG; right) electrodes. Cyan electrodes denote stimulation pairs (bipolar pulses; 10 mA, 500 micro-second pulse width; 1 Hz for 50s). Amplitude (radius of electrode) and latency (color) of the N1 responses are represented. Electrodes without CCEP responses are depicted as white spheres. Normalized evoked potentials (waveform deflections) are plotted for the encircled electrodes recording from (left inset) f-IOG and f-FG electrodes and (right inset) EVC and f-FG electrodes. Shadings represent 1 SEM (n = 50 stimulation trials). (Bottom) Scatter & box plots for all subjects that underwent CCEP recordings during either EVC stimulation or f-FG stimulation. Points represent single-trial N1 latency differences between f-IOG and f-FG electrodes during EVC stimulation (left), and between EVC and f-IOG electrodes during f-FG stimulation (right).

Stimulations at electrodes localized over the EVC were performed in 3 subjects (Fig 5 *left*; RH–subject 4; LH–subjects 5 and 7). N1 latencies were identified at the single trial level (i.e. for each stimulation pulse; *n* = 50 trials per stimulation session) for each f-IOG or f-FG electrode per subject. Median N1 latency differences were then computed for each subject by performing single-trial, pair-wise contrasts between all pairs of f-IOG and f-FG electrodes. This difference value is negative if the f-IOG N1 precedes that in the f-FG, and positive if the reverse is true. Computing differences values in this fashion accounts for the natural variation in N1 latencies across different subjects. Subject 4 underwent stimulation at 2 pairs of EVC electrodes with concurrent icEEG recording at 3 f-IOG and 2 f-FG electrodes, leading to a total of 6 sets of paired electrode contrasts. Each paired-contrast provided 100 difference values (50 trials per each EVC stimulation session), leading to a total of 600 difference values (6 paired contrasts each with 100 difference values). The mean (sd) latency difference for all 600 trials in Subject 4 was 2.68 (11.04) ms. Subject 5 underwent stimulation at 3 EVC pairs (150 trials/paired contrast) with concurrent icEEG recording at 3 f-IOG and 3-fFG electrodes (9 paired contrasts), and a mean (sd) latency difference of -2.51 (11.18) ms (n = 1350 total trials). And subject 7 underwent stimulation at 3 EVC pairs (150 trials/paired contrast) with concurrent icEEG recordings at 1 f-IOG and 3 f-FG electrodes (3 paired contrasts), and a mean (sd) latency difference of -1.71 (10.77) ms (n = 450 total trials). Overall, across the 3 subjects that underwent EVC stimulation, no significant differences in N1 latencies were found between f-IOG and f-FG electrodes (mean [sd]: -1.1 [3.01] ms; *p* = 0.547; two-sided, non-parametric Wilcoxon sign-rank test).

Stimulation at electrodes localized over the f-FG was performed in 5 subjects (Fig 5 *right*; RH–subjects 1, 2, 4; LH–subjects 5 and 7). During each stimulation session, N1 latencies were identified at the single trial level (i.e. for each stimulation pulse; *n* = 50 trials per stimulation session) for each EVC or f-IOG electrode per subject. Median N1 latency differences were then computed for each subject by performing single-trial pair-wise contrasts between all pairs of EVC and f-IOG electrodes. This difference value is negative if the EVC N1 precedes that in the f-IOG, and positive if the reverse is true. Subject 1 underwent stimulation at 1 pair of f-FG electrodes (50 trials/paired contrast) with concurrent recordings at 4 EVC and 2 f-IOG electrodes (8 paired contrasts), and a mean (sd) latency difference of -2.25 (9.86) ms (n = 400 trials). Subject 2 underwent stimulation at 2 f-FG pairs (100 trials/paired contrast) with concurrent recordings in 3 EVC and 3 f-IOG electrodes (9 paired contrasts), for a mean (sd) latency difference of -1.63 (11.09) ms (n = 900 total trials). Subject 4 underwent stimulation at 1 f-FG pair (50 trials/paired contrast) with concurrent icEEG at 3 EVC and 3 f-IOG electrodes (9 paired contrasts), for a mean (sd) latency difference of -6.26 (11.21) ms (n = 450 total trials). Subject 5 underwent stimulation at 1 f-FG pair (50 trials/paired contrast) with concurrent icEEG recorded at 3 EVC and 3 f-IOG electrodes (9 paired contrasts), for a mean (sd) latency difference of 1.09 (10.35 ms) (n = 450 total trials). Lastly, subject 7 underwent stimulation at 1 f-FG pair (50 trials/paired contrast) with concurrent icEEG recorded at 8 EVC and 1 f-IOG electrode (8 paired contrasts), for a mean (sd) latency difference of -1.87 (10.33) ms (n = 400 total trials). Overall, across the 5 subjects that underwent f-FG stimulation, no significant differences in N1 latencies were found between EVC and f-IOG electrodes (mean [sd] N1 latency difference: -2.15 [3.14] ms; *p* = 0.156; two-sided, non-parametric Wilcoxon sign-rank test).

## Discussion

We used high spatiotemporal resolution icEEG recordings and cortical stimulation in both hemispheres to evaluate serial and parallel models of information flow within the core face network during a face-naming task. Our work suggests that current serial accounts of invariant face perception [1, 42, 57] are likely incorrect based on three distinct findings: (1) the onset of face selectivity is not significantly different between the f-IOG and f-FG in the right hemisphere; (2) feedforward connectivity from right EVC to f-FG precedes bidirectional, task-dependent changes in connectivity between f-IOG and f-FG; and (3) signal propagation latencies between EVC and f-IOG are not significantly faster than between EVC and f-FG. Taken together, these findings are more consistent with parallel network models of invariant face perception [3, 4].

Given the f-IOG’s posterior location, the serial, hierarchical model of face perception[1] implicitly assumes that low-level visual information from the EVC input first reaches the f-IOG, which initiates a parts-based analysis prior to relaying information to the f-FG for structural encoding [3, 42, 57]. If the serial model is correct, unidirectional and feed-forward connectivity between the EVC and f-IOG is expected to appear prior to feedforward connectivity between f-IOG and f-FG. Moreover, f-IOG should be the first region to demonstrate face-selective activity [46]. In contrast to these predictions, our findings reveal that face-selectivity onsets are coincident between f-IOG and f-FG in the right hemisphere, and that feed-forward interactions between right EVC and f-FG lead task-dependent, bidirectional changes in the interactions between right f-IOG and f-FG. These results are more consistent with parallel network model predictions, in which independent EVC input enables the early detection of faces by the f-FG, following which the f-IOG and f-FG work together using re-entrant connections to refine facial features [4]. Importantly, the demonstration of short-latency, bidirectional N1 responses between these three regions independently validates the presence of the parallel cortico-cortical pathways needed to mediate such rapid information flow between these three regions [89–91, 104]. These CCEP findings are supported by human tractography studies demonstrating direct white-matter connections between the EVC and these two regions [104–108], as well as by a recent anatomical tracer study performed in functionally-defined monkey face patches, which revealed a dense, patch-specific network of parallel feedforward and feedback connections[18]. Crucially, the conclusions from this macaque study also directly challenged the serial model of ventral visual face processing (e.g. f-IOG to f-FG), arguing for a parallelized network organization that is much more analogous to the parallel model evaluated here[18].

Our time-series and connectivity analyses further revealed interesting differences between right and left hemisphere network behavior. Specifically, while face-selectivity onsets were comparable between the right f-IOG and f-FG, face-selectivity in the left f-IOG typically preceded those in the left f-FG. Furthermore, in the left hemisphere, baseline connectivity was largely absent, while early post-stimulus (<100ms) interactions were only noted between f-IOG and f-FG. These findings contrast with the right hemisphere, in which baseline interactions extended well beyond stimulus onset between all three regions pairs. We note, however, that the network dynamics observed in the left hemisphere are nevertheless inconsistent with serial model predictions—specifically in the onsets of left EVC⇒f-FG and then EVC⇒f-IOG connectivity, both of which emerge subsequent to f-IOG⇒f-FG interactions. The nature of these differences, and their functional consequence to face processing, will inform future work.

The hemispheric distinctions in our findings–observed in the coincident onsets of right (but not left) hemispheric face-selectivity and the earlier patterns of right hemispheric functional connectivity (<100 ms)–reinforce the right hemispheric bias and importance of the right fusiform gyrus for face perception [2, 4, 25, 26, 32, 80]. Given the timing at which early EVC⇒f-FG connectivity begins (<100 ms), face-detection in the right hemisphere likely occurs as an automatic process prior to conscious perception [53, 102, 109]. These early interactions may also reflect the predictive coding and/or expectation bias of higher visual cortical regions, which may facilitate the perceptual processing of preferred stimuli[110–112]. Similarly, the unique patterns of early (<~100 ms) f-IOG⇔f-FG connectivity support prior reports of strong f-IOG⇔f-FG resting-state correlations that are modulated by preferred stimuli (e.g. faces) in a task-dependent fashion [113, 114] [115]. Interestingly, the subsequent re-emergence of feed-forward EVC connectivity, with both the f-IOG and f-FG at ~100 ms, precedes task-related increases in BGA power in these two core face regions. The intense, task-dependent onset in broadband gamma activity is believed to reflect a rapid and large increase in regional neural activity, which mediates higher-level face processing and is coupled to perceptual awareness [52, 78]. Taken together, these results would then implicate EVC input as the match that “ignites” perceptual face processing in these core face-regions [52]. In contrast, task-dependent changes in f-IOG⇔f-FG connectivity (~150 ms) coincide with the emergence of face-selective activity from both f-IOG and f-FG time series (~120–200 ms). Taken with the (relatively) later onset and bidirectional nature of f-IOG⇔f-FG connectivity, this suggests that the relevant visual information is already available in both of these regions, following which reentrant interactions become more prominent as higher-level face processing through feature refinement proceeds[103, 116].

In conclusion, we integrate measures of cortical activation, functional and electrophysiological connectivity to demonstrate that the neural mechanisms that underpin face perception cannot be adequately explained by current serial accounts[3]. Rather, the core face-network appears to operate in a parallel, distributed manner [4]. Inherent limitations of invasive studies in humans—small subject numbers, sites of electrode placements and stimulation parameters determined by clinical rather than research criteria—preclude a more comprehensive validation of predictions made by parallel network accounts of face perception. Furthermore, our findings cannot rule out the possibility that face perception may invoke both serial and parallel operations[117], even differentially across hemispheres. Our results may also not be relevant beyond the visual naming paradigm that we have tested, as face processing involves complex interactions across many more cortical regions than the three investigated here. However, our findings do generate specific predictions regarding the timing and regional interactions of critical stages of face-perception, which can be validated through chronometric or real-time stimulation by future studies. And while we cannot categorically prove that the activation patterns evaluated here are related to face-perception, icEEG recordings do enable us to track stimulus-induced changes in cortical activity with millisecond temporal resolution. Given that we do observe significantly greater activations to face stimuli (vs. low and high-level non-face control stimuli), which emerge ~150 to 200 ms after image presentation, and are localized within in higher-level ventral temporal regions known to play critical roles in face perception, it seems reasonable to assume that such activity is related to face perception[103].

There are a number of limitations of this work that are inherent to icEEG studies in humans: Small sample sizes and sparsely distributed recordings, which derive from the invasive nature of electrode implantation and need to minimize risk. This may seem to under-power our analyses, especially with respect to face-selectivity onset latencies in which the results (often in the millisecond range) depend on detecting differences across few numbers of subjects. However, icEEG data are collected at millisecond or sub-millisecond sampling rates, and by utilizing paired-electrode contrasts to evaluate within-subject timing differences (e.g. face-selectivity onset latencies), we have nevertheless been able to demonstrate significant findings between the f-IOG and f-FG (e.g. in our left hemispheric cohort) despite the relatively low number of subjects in this study. Such results increase our confidence that our data do indeed have the requisite temporal resolution to detect the presences or absence of such differences. Ultimately, however, limitations related to small sample sizes can only truly be overcome by integrating data across multiple subjects. For the current study, concurrent coverage over all three posterior visual regions of interest (EVC, f-IOG, and f-FG) was required. The occurrence of such coverage is quite rare, however, due to the low frequency with which focal epilepsy originates in visual regions (data for this study were obtained over the course of 7 years). Once acquired, however, icEEG’s unique spatiotemporal resolution offers an unparalleled opportunity to study human cognition that remains out of reach for current non-invasive neuroimaging approaches (e.g. fMRI) [118]. The evaluation of the nature of transient interactions between difficult-to-access regions in higher-level visual cortex, as in this case, reflects just one such example.

A second, related, limitation of icEEG (also because of patient safety considerations) is the sparse-coverage problem [70, 118]. Given the clinically guided implantation of electrodes, it is unavoidable that electrodes will not all be localized within the same exact sections of EVC, IOG, and FG across subjects. This issue is further complicated because the variable folding of human cortex precludes the perfect alignment of homologous functional regions across subjects[70]. A serious concern that results from these complications is whether the inter-subject anatomical variability, both in cortical anatomy as well as relative electrode location, limits interpretability of our results (e.g. with respect to timing contrasts). More specifically, if we have both sparse sampling of regions and the exact same pairs of neuronal sites are not sampled across subjects, how does that impact our ability to draw conclusions regarding timing differences? Our approach to addressing this issue was to utilize multiple, strict anatomical and functional criteria to ensure that any electrodes included in the analyses were recording from functionally homologous regions of interest across subjects. These anatomical criteria incorporate surface-based algorithms that take into consideration both gyral and sulcal cortical folding patterns to maximize topological accuracy. As a result, any electrode included in this analysis was one that was ultimately been determined to 1) be localized over anatomically-defined EVC, IOG, or lateral FG, and 2) demonstrate functional response properties consistent with either EVC or face-selective regions of the IOG and FG. By ensuring that the selected electrodes are localized within well-defined anatomical boundaries, and that recorded cortical activity patterns are consistent with the known functional role of each region, we are able to best ensure that our findings are relevant for the cortical networks we hope to investigate.

In contrast to the task-dependent nature of icEEG, CCEPs provide a task-independent metric for the study of cortical network organization. Nevertheless, CCEPs also suffer several limitations. The first is that current injection at the stimulation site produces a 2–5 ms long stimulus artifact that could mask mono- or di-synaptic cortico-cortical connections. Second, the electrophysiology and circuit-level mechanisms that characterize the evoked N1 still remain poorly characterized. Future studies on the relationship between electrode configuration (e.g. its orientation to underlying cortical columns) and the properties of the evoked potential (e.g. latency, polarity, and strength; orthodromic or antidromic activation of axons) will be necessary to better answer these questions. Finally, the variability in stimulation design (electrode type, current amplitude, pulse duration, ISI, numbers of repetitions, etc.) and patient cognitive states (e.g. anesthetized/sedated vs. awake/medicated) make it difficult to critically evaluate findings across different study centers. However, arguments favoring the generalization of findings across patients and study centers are supported by the overall consistency of results across patients and centers[89].

A final, important caveat is that our icEEG and CCEP analyses ultimately provide only indirect evidence in evaluating different network models of face perception. Therefore, they cannot be used to conclusively prove or disprove one model over the other. By contrast, the transient disruption of local function, using cortical stimulation mapping, provides an important opportunity to directly and causally evaluate specific model predictions [68]. Notably, while the existent literature on invasive f-IOG macro-stimulation derives entirely from a single subject [119, 120], both studies reported on this subject indicate that macro-stimulation of the f-IOG caused a disruption only in this subject’s ability to *individuate* different faces–in contrast to serial model predictions. These findings were strongly reinforced by the most recent publication from this group, which demonstrated that the complete resection of the right inferior occipital cortex in this subject (including the entirety of the f-IOG) did not disrupt the longitudinal stability (over 8 months) of downstream ipsilateral face-selective regions [117]. Tractography analysis performed both before and after resection further revealed persistent white matter connections between early visual retinotopic areas and these downstream face-selective regions[117]. Similarly, non-invasive stimulation studies of the f-IOG, using transcranial magnetic stimulation have reported reduced accuracy rates during individuation tasks, but not basic-level face categorization [41, 42]. Finally, prior cortical stimulation studies in the right (but not left) f-FG have consistently produced disruptions to the earliest-stages of face perception [68, 80, 121–123]. Taken together, these differential effects provide strong, causal support to implicate cortical substrates in the right f-FG, but not f-IOG, as the neural circuitry most critical to face-perception [25, 80, 120].

In sum, our results add to a growing body of literature that implicate higher visual areas as active participants in object processing [48, 81, 124], consistent with predictive coding, reverse-hierarchical, and top-down interpretations of visual recognition [110, 112, 125]. Our findings also highlight the need to critically evaluate existing and future cognitive network models using both cortical activity and inter-areal connectivity captured at sufficient spatio-temporal resolution. Improvements in our ability to accurately model cognitive function will have important implications for understanding and developing treatments for disease states, such as prosopagnosia, that arise from the disruption of these complex networks.

## Supporting information

S1 Fig Significance and correlation plots of grouped functional connectivity. *(A)* Significance plots: Onset and time-course of significant correlations (*q*<0.01) between region pairs from the grouped AEC results (Fig 4) for all feed-forward time-lags (0 to +150ms). (*Center)* Cross-correlogram template (used in Fig 4) depicts how feed-forward (i.e. positive) time-lags are plotted in relation to the data. Each time-lag progresses along a different diagonal above the black dashed diagonal (indicating 0ms lag). Gray shadings used to visually distinguish two specific feed-forward lags (+75ms and +150ms) on the correlogram, and match time-lags on the significance plots. Gray shadings do not indicate an average or binning of time-lags. (*Left)* Significance plots for grouped AEC between the three region pairs in the right hemisphere: EVC–to–f-IOG (*top box*, *green*), EVC–to–f-FG (*middle box*, *red*), and f-IOG–to–f-FG (*bottom box*, *blue*). Colored bars indicate the presence of significant correlations (i.e. contour lines in Fig 4) between a given region pair at a specific time and lag. X-axis depicts the timeline (0 to 250ms after stimulus onset) for the region named first (e.g. EVC’s timeline in the EVC to f-IOG box). Y-axis indicates the time-lag for the correlations computed with the second region (e.g. for f-IOG in the EVC to f-IOG box). Gray shadings along the Y-axis match gray shadings on the center cross-correlogram. (*Right)* Significance plots for the grouped AEC results between the three region pairs in the left hemisphere. *(B)* Correlation plots: Time-lagged correlations between regions plotted for a single lag value (*top* +75ms; *bottom* +150ms*)*. EVC⇒f-IOG (*green*), EVC⇒f-FG *(red)*, and f-IOG⇒f-FG traces (*blue)* reflect correlational values along a single diagonal (+75 or +150ms lag) from the grouped AEC cross-correlograms (in Fig 4). Shadings denote 1 SEM (across subjects; RH *n = 3*; LH *n* = 5). Solid bars below traces depict onset of significant connectivity (*q<0*.*01;* color-coded by region pair), and are equivalent to a single horizontal line from Significance Plots (*above*). Notably, in the right hemisphere (*left plot)*, increases in the magnitude of significant EVC⇒f-FG connectivity at ~100ms (red trace and solid bar, respectively) precede changes in significant f-IOG⇒f-FG connectivity (blue trace and bar, respectively).
(TIF)

S2 Fig Non-smoothed functional connectivity during face perception. *(A) Note: Analyses in this figure are identical to Fig 4, with the exception that the BGA amplitude envelopes were not smoothed prior to the computation of amplitude envelop correlations. Instead, correlations were performed using 5 ms, non-overlapping intervals, in order to confirm that envelope smoothing did not introduce false correlations.* Group temporal cross correlograms of right hemisphere EVC-f-IOG connectivity, computed by averaging individual amplitude envelope correlations (5ms time bins; *n* = 3 subjects, contours denote significant connectivity, *q* = 0.05, FDR corrected) for face stimuli only. Amplitude envelope correlations are measured across lag ranges of -150 to +150 ms. The black dashed diagonal line represents a lag of 0 ms. Above the dashed line activity in EVC activity leads f-IOG (information flow from EVC to the f-IOG), while below the dashed line f-IOG activity leads EVC (information flow from f-IOG to EVC). *(B)* Connectivity between EVC and the f-FG, right hemisphere (*n* = 3 subject). *(C)* Connectivity between f-IOG and f-FG, right hemisphere (*n* = 3 subject). *(D)* Connectivity between EVC and the f-IOG, left hemisphere (*n* = 4 subject). *(E)* Connectivity between EVC and the f-FG, left hemisphere (*n* = 5 subject). *(F)* Connectivity between f-IOG and the f-FG, left hemisphere (*n* = 4 subject).
(TIF)

S3 Fig Variability in location of peak f-IOG and f-FG face-selectivity in grouped healthy subject fMRI. *NOTE*: *Spheres here denote the location of peak fMRI activation for individual healthy subjects for the face localizer task (not electrodes)*. Each sphere denotes the highest fMRI activation in the f-IOG and f-FG face selective clusters (faces > animate, inanimate, and scramble; p <0.01) from each of the 18 individual healthy subjects that contributed to the grouped fMRI dataset. The spheres are visualized together on one template anatomy (MNI N27 template brain aligned to Talairach coordinate space) to visually depict the degree of inter-subject fMRI variability between hemispheres. The results of the grouped fMRI analysis from the same 18 healthy volunteers are also depicted on the cortical surface, which have been co-registered to this template anatomy using a surface-based normalization technique.
(TIF)

S1 Data LH EVC icEEG data: Matlab structure containing icEEG for each LH subject with EVC electrodes, with fields for normalized percent change, mean baseline, standard deviation of baseline, and normalized power envelopes of BGA. Data for all icEEG tasks are provided (faces/proper names; tools; non-tools; animate; and scrambled). The S2–S6 Datasets are organized in the same fashion.
(MAT)

S2 Data LH LOC icEEG data. (MAT)

S3 Data LH VTC icEEG data. (MAT)

S4 Data RH EVC icEEG data. (MAT)

S5 Data RH LOC icEEG data. (MAT)

S6 Data RH VTC icEEG data. (MAT)

S7 Data Bilateral CCEP data. Trial-by-trial difference values for subjects that underwent CCEP recordings in both EVC and f-FG sessions.
(MAT)
