# Supplementary figures and images for: Network dynamics of human face perception
Source: PLoS One. 2017 Nov 30;12(11):e0188834. doi: 10.1371/journal.pone.0188834 (PMC5708727; doi:10.1371/journal.pone.0188834)

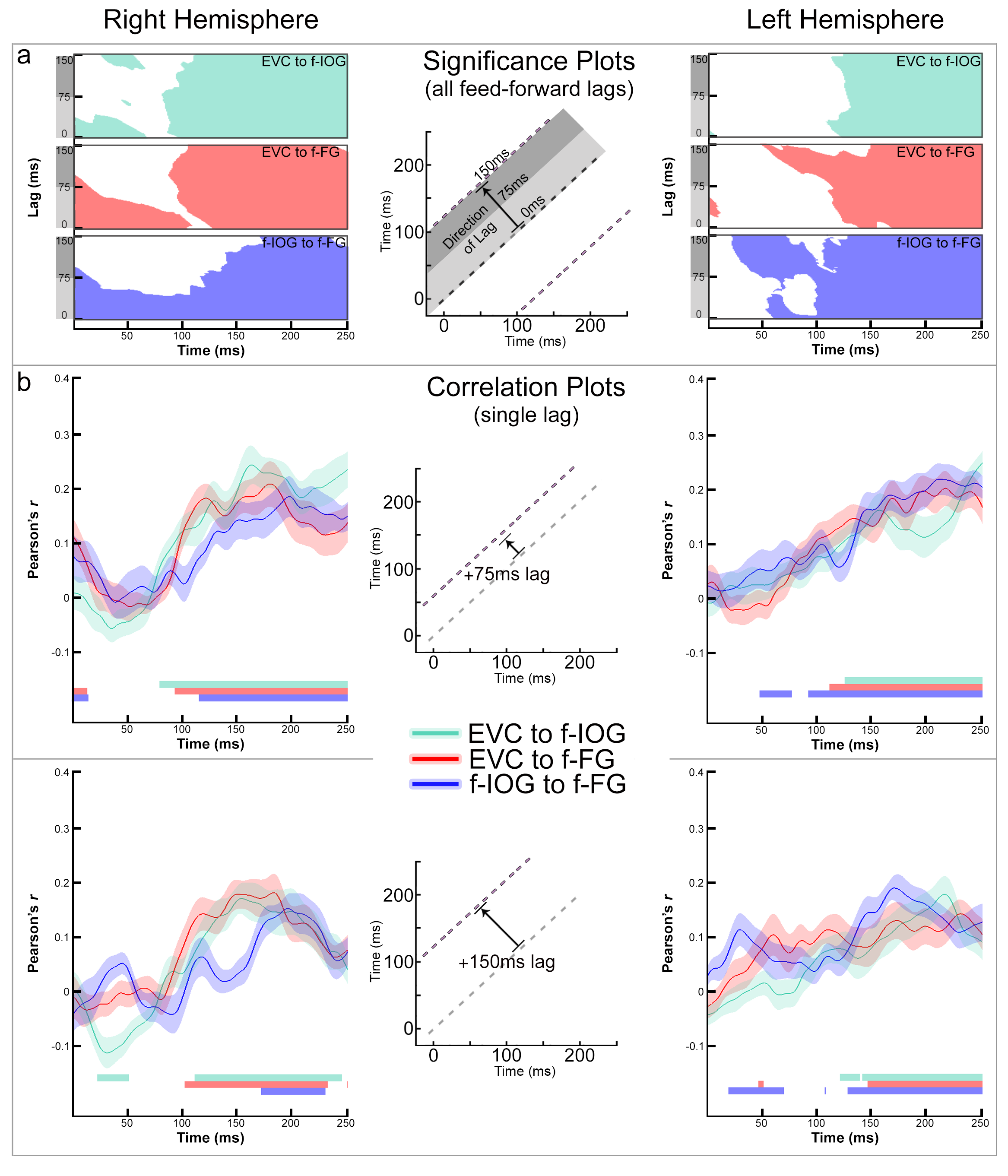

Supplement: S1 Fig — (A) Significance plots: Onset and time-course of significant correlations (q<0.01) between region pairs from the grouped AEC results (Fig 4) for all feed-forward time-lags (0 to +150ms). (Center) Cross-correlogram template (used in Fig 4) depicts how feed-forward (i.e. positive) time-lags are plotted in relation to the data. Each time-lag progresses along a different diagonal above the black dashed diagonal (indicating 0ms lag). Gray shadings used to visually distinguish two specific feed-forward lags (+75ms and +150ms) on the correlogram, and match time-lags on the significance plots. Gray shadings do not indicate an average or binning of time-lags. (Left) Significance plots for grouped AEC between the three region pairs in the right hemisphere: EVC–to–f-IOG (top box, green), EVC–to–f-FG (middle box, red), and f-IOG–to–f-FG (bottom box, blue). Colored bars indicate the presence of significant correlations (i.e. contour lines in Fig 4) between a given region pair at a specific time and lag. X-axis depicts the timeline (0 to 250ms after stimulus onset) for the region named first (e.g. EVC’s timeline in the EVC to f-IOG box). Y-axis indicates the time-lag for the correlations computed with the second region (e.g. for f-IOG in the EVC to f-IOG box). Gray shadings along the Y-axis match gray shadings on the center cross-correlogram. (Right) Significance plots for the grouped AEC results between the three region pairs in the left hemisphere. (B) Correlation plots: Time-lagged correlations between regions plotted for a single lag value (top +75ms; bottom +150ms). EVC⇒f-IOG (green), EVC⇒f-FG (red), and f-IOG⇒f-FG traces (blue) reflect correlational values along a single diagonal (+75 or +150ms lag) from the grouped AEC cross-correlograms (in Fig 4). Shadings denote 1 SEM (across subjects; RH n = 3; LH n = 5). Solid bars below traces depict onset of significant connectivity (q<0.01; color-coded by region pair), and are equivalent to a single horizontal line from Significanc [file pone.0188834.s001.tif]

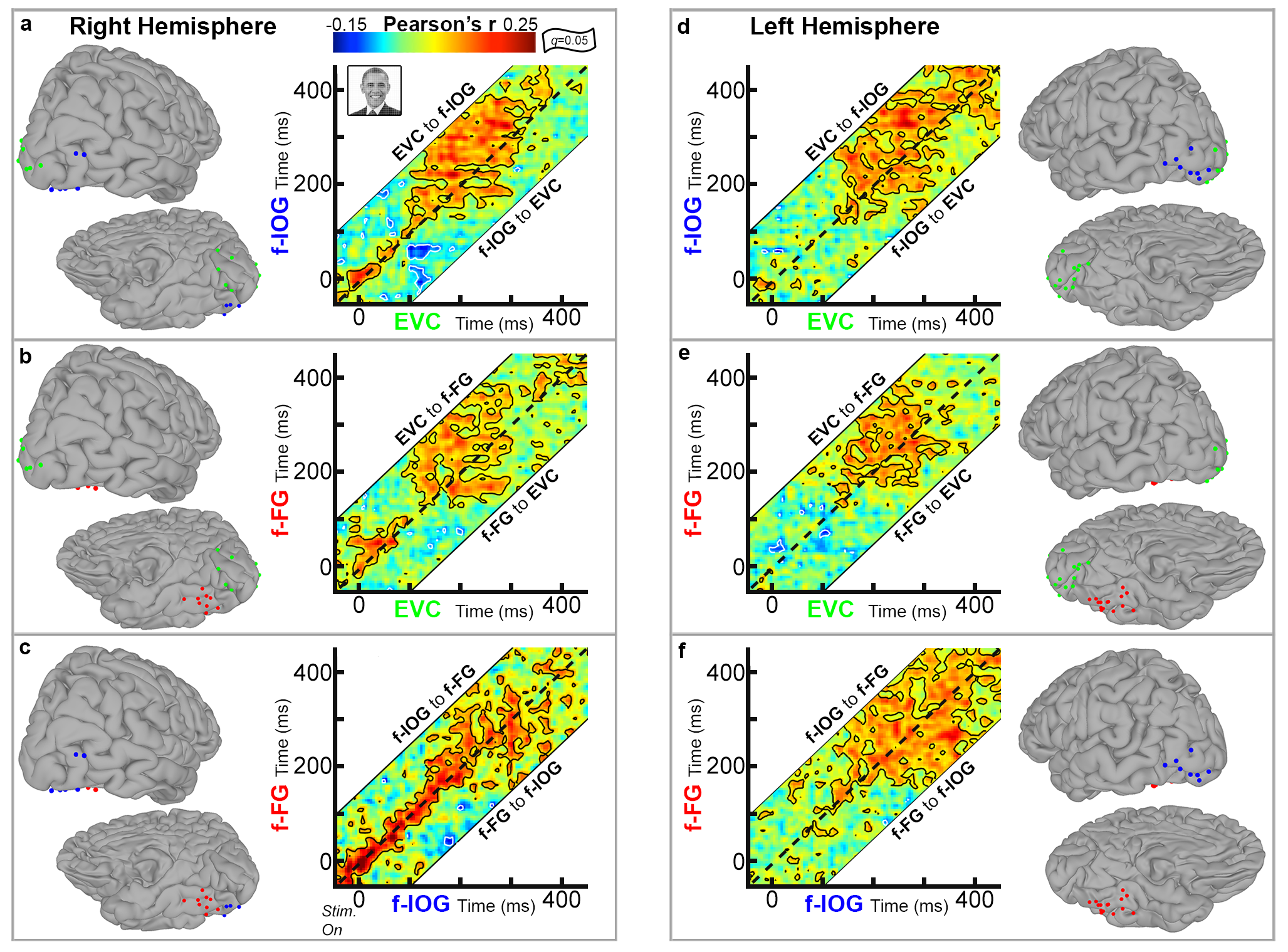

Supplement: S2 Fig — (A) Note: Analyses in this figure are identical to Fig 4, with the exception that the BGA amplitude envelopes were not smoothed prior to the computation of amplitude envelop correlations. Instead, correlations were performed using 5 ms, non-overlapping intervals, in order to confirm that envelope smoothing did not introduce false correlations. Group temporal cross correlograms of right hemisphere EVC-f-IOG connectivity, computed by averaging individual amplitude envelope correlations (5ms time bins; n = 3 subjects, contours denote significant connectivity, q = 0.05, FDR corrected) for face stimuli only. Amplitude envelope correlations are measured across lag ranges of -150 to +150 ms. The black dashed diagonal line represents a lag of 0 ms. Above the dashed line activity in EVC activity leads f-IOG (information flow from EVC to the f-IOG), while below the dashed line f-IOG activity leads EVC (information flow from f-IOG to EVC). (B) Connectivity between EVC and the f-FG, right hemisphere (n = 3 subject). (C) Connectivity between f-IOG and f-FG, right hemisphere (n = 3 subject). (D) Connectivity between EVC and the f-IOG, left hemisphere (n = 4 subject). (E) Connectivity between EVC and the f-FG, left hemisphere (n = 5 subject). (F) Connectivity between f-IOG and the f-FG, left hemisphere (n = 4 subject). (TIF) [file pone.0188834.s002.tif]

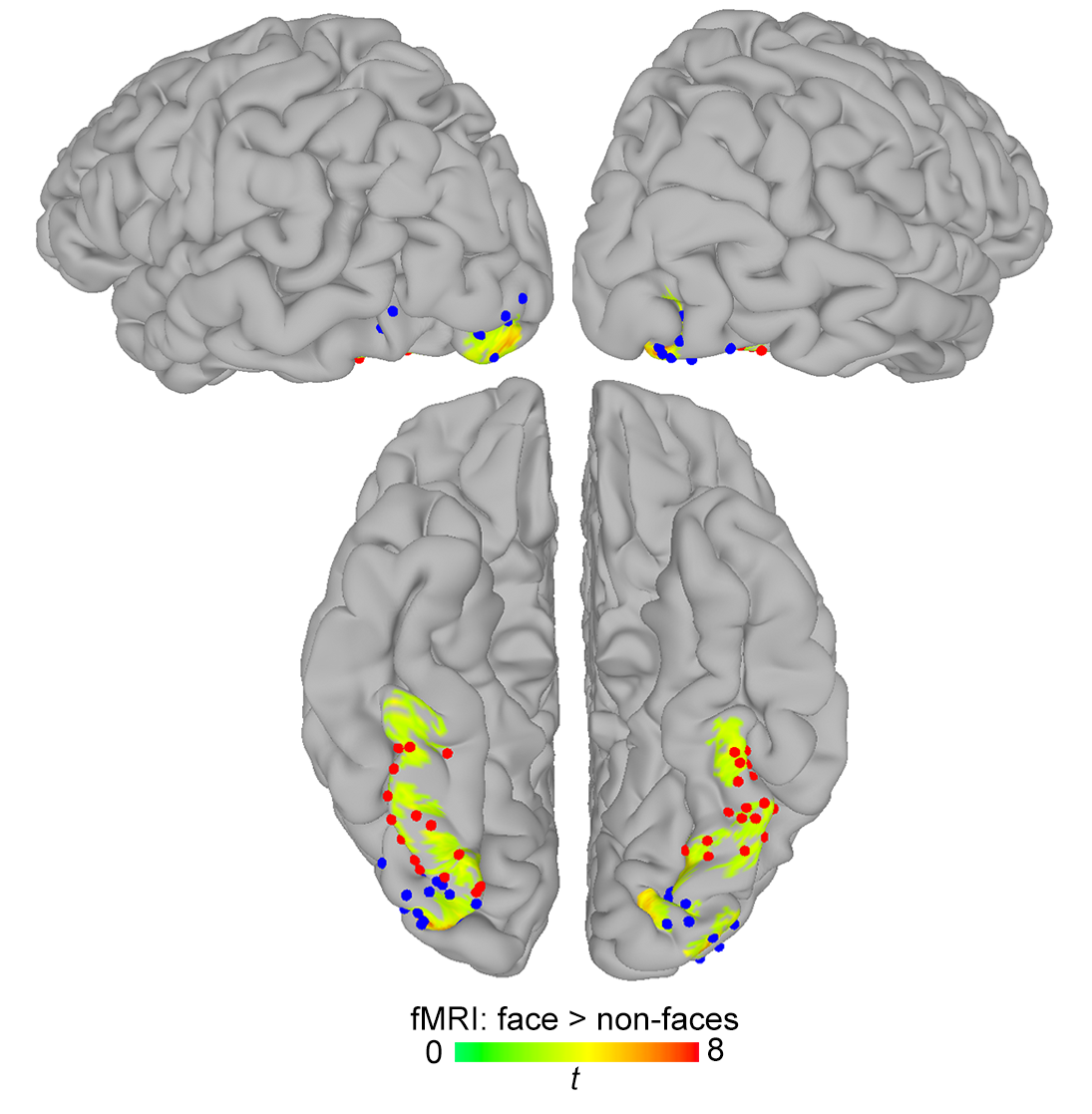

Supplement: S3 Fig — NOTE: Spheres here denote the location of peak fMRI activation for individual healthy subjects for the face localizer task (not electrodes). Each sphere denotes the highest fMRI activation in the f-IOG and f-FG face selective clusters (faces > animate, inanimate, and scramble; p <0.01) from each of the 18 individual healthy subjects that contributed to the grouped fMRI dataset. The spheres are visualized together on one template anatomy (MNI N27 template brain aligned to Talairach coordinate space) to visually depict the degree of inter-subject fMRI variability between hemispheres. The results of the grouped fMRI analysis from the same 18 healthy volunteers are also depicted on the cortical surface, which have been co-registered to this template anatomy using a surface-based normalization technique. (TIF) [file pone.0188834.s003.tif]
